# Gaussian graphical modeling reconstructs pathway reactions from high-throughput metabolomics data

**DOI:** 10.1186/1752-0509-5-21

**Published:** 2011-01-31

**Authors:** Jan Krumsiek, Karsten Suhre, Thomas Illig, Jerzy Adamski, Fabian J Theis

**Affiliations:** 1 Institute of Bioinformatics and Systems Biology, Helmholtz Zentrum München, Germany; 2 Faculty of Biology, Ludwig-Maximilians-Universität, Planegg-Martinsried, Germany; 3 Institute of Epidemiology, Helmholtz Zentrum München, Germany; 4 Institute of Experimental Genetics, Genome Analysis Center, Helmholtz Zentrum München, Germany; 5 Department of Mathematics, Technische Universität München, Germany

## Abstract

**Background:**

With the advent of high-throughput targeted metabolic profiling techniques, the question of how to interpret and analyze the resulting vast amount of data becomes more and more important. In this work we address the reconstruction of metabolic reactions from cross-sectional metabolomics data, that is without the requirement for time-resolved measurements or specific system perturbations. Previous studies in this area mainly focused on Pearson correlation coefficients, which however are generally incapable of distinguishing between direct and indirect metabolic interactions.

**Results:**

In our new approach we propose the application of a Gaussian graphical model (GGM), an undirected probabilistic graphical model estimating the conditional dependence between variables. GGMs are based on partial correlation coefficients, that is pairwise Pearson correlation coefficients conditioned against the correlation with all other metabolites. We first demonstrate the general validity of the method and its advantages over regular correlation networks with computer-simulated reaction systems. Then we estimate a GGM on data from a large human population cohort, covering 1020 fasting blood serum samples with 151 quantified metabolites. The GGM is much sparser than the correlation network, shows a modular structure with respect to metabolite classes, and is stable to the choice of samples in the data set. On the example of human fatty acid metabolism, we demonstrate for the first time that high partial correlation coefficients generally correspond to known metabolic reactions. This feature is evaluated both manually by investigating specific pairs of high-scoring metabolites, and then systematically on a literature-curated model of fatty acid synthesis and degradation. Our method detects many known reactions along with possibly novel pathway interactions, representing candidates for further experimental examination.

**Conclusions:**

In summary, we demonstrate strong signatures of intracellular pathways in blood serum data, and provide a valuable tool for the unbiased reconstruction of metabolic reactions from large-scale metabolomics data sets.

## Background

Metabolomics is a newly arising field aiming at the measurement of all endogenous metabolites of a tissue or body fluid under given conditions [1-3]. The resulting *metabolome *of a biological system is considered to provide a readout of the integrated response of cellular processes to genetic and environmental factors [4]. Understanding the complex biochemical interplay between hundreds of measured metabolite species is a daunting task, which can be approached by combining advanced computational methods with data from large population-based studies. On the biochemical level, metabolite concentrations are determined by a set of specific metabolic enzymes. Variabilities in both enzyme activity and metabolite exchange rates - induced by a continuous spectrum of metabolic states throughout measured samples - give rise to characteristic patterns in the metabolite profiles which are directly linked the underlying biochemical reaction network [5,6]. Although human metabolism has been extensively characterized in the past decades [7], the reconstruction of metabolic networks from such metabolite patterns is a key question in the computational research field. Previous attempts focused on linear metabolite associations measured by Pearson correlation coefficients. These include studies utilizing time-course measurements and clustering [8], theoretical approaches relating metabolite fluctuations to properties of the dynamical system [5] and metabolic control analysis to derive effects of enzyme variability [6]. Other reconstruction methods rely on specific perturbations of the biological system, like the induction of concentration pulses for certain metabolites [9].

A major drawback of correlation networks, however, is their inability to distinguish between direct and indirect associations. Correlation coefficients are generally high in large-scale *omics *data sets, suggesting a plethora of indirect and systemic associations. For example, transcriptional coregulation amongst many genes will give rise to indirect interaction effects in mRNA expression data [10]. Similar effects can be observed in metabolic systems which, in contrast to genetic networks, contain fast biochemical reactions in an open mass- flow system. Metabolite levels are supposed to be in quasi-steady state compared to the time scales of upstream regulatory processes [11]. That is, metabolites will follow changes in gene expression and physiological processes on the order of minutes and hours, but will appear unchanged on the order of seconds. These properties, even though substantially different from mRNA expression mechanisms, also give rise to indirect, system-wide correlations between distantly connected metabolites.

*Gaussian graphical models *(GGMs) circumvent indirect association effects by evaluating *conditional *dependencies in multivariate Gaussian distributions [10]. A GGM is an undirected graph in which each edge represents the pairwise correlation between two variables conditioned against the correlations with all other variables (also denoted as *partial *correlation coefficients). GGMs have a simple interpretation in terms of linear regression techniques. When regressing two random variables *X *and *Y *on the remaining variables in the data set, the partial correlation coefficient between *X *and *Y *is given by the Pearson correlation of the residuals from both regressions. Intuitively speaking, we remove the (linear) effects of all other variables on *X *and Y and compare the remaining signals. If the variables are still correlated, the correlation is directly determined by the association of X and Y and not mediated by the other variables. Partial correlations have recently been applied to biological data sets for the inference of association networks from mRNA expression data [12-15], and for the elucidation of relationships between genomic features in the human genome [16]. One previous study used second-order partial correlations of genetic associations to elucidate genetically determined relations between metabolites [17].

In this manuscript we now study the capabilities of GGMs to recover metabolic pathway reactions solely from measured metabolite concentrations. First, we discuss the quality of the method and possible problems and pitfalls on computer-simulated systems. We then apply GGMs to a lipid-focused targeted metabolomics data set of 1020 blood serum samples with 151 measured metabolites from the German population study KORA [18,19]. The GGM is sparse in comparison to the corresponding Pearson correlation network, displays a modular structure with respect to different metabolite classes, and is stable towards changes in the underlying data set. We demonstrate that top-ranking metabolite pairs and further densely connected subgraphs in the GGM can indeed be attributed to known reactions in the human fatty acid biosynthesis and degradation pathways. In order to systematically verify this finding, we map partial correlation coefficients to the number of reaction steps between all metabolite pairs based on a literature-curated fatty acid pathway model. We observe statistically significant discriminatory features of GGMs to distinguish between directly and non-directly interacting metabolites in the metabolic network. In addition, low-order partial correlations turned out to be a suitable alternative to full-order GGMs for the present dataset. Finally, we will summarize and discuss the relevance of GGMs for metabolomics data sets, point out limitations of the method and suggest future steps. All metabolomics data used in this study, the generated correlation networks, model files and metabolite annotations are available online at http://hmgu.de/cmb/ggm.

## Results and Discussion

### GGMs delineate direct relationships in artificial reaction systems

Computer-simulated reaction systems are a valuable tool for the evaluation of correlation-based measures prior to their application to real metabolomics data sets. Previous works focused on the modeling of biological replicates with intrinsic noise on the metabolite levels [5]. In contrast, we here investigate the effects of variation of enzymatic activity in a human population cohort. Such variation might be genetically determined or, more likely, be the result of distinct regulatory effects and metabolic states between individuals. All reaction systems were implemented as ordinary differential equations with simple mass-action kinetics rate laws and reversible Michaelis-Menten-type enzyme kinetics (see Methods). In order to account for the above-mentioned enzymatic variability we applied a log-normal noise model, which has been previously described to be a reasonable approximation of cellular rate parameter distributions [20]. The standard deviation *σ *was set to a value of 0.2 for the underlying normal distribution (note that the results are insensitive to the magnitude of *σ*). For each parameter sample we calculated the metabolite steady state concentrations on log-scale, and subsequently estimated the GGM by calculating partial correlation coefficients. All analyzed systems exhibit single, unique steady states independent of the respective parameter values. This feature was structurally verified using the ERNEST toolbox [21] for all networks except the negative feedback system. For the latter one, we employed empirical initial state sampling to ensure monostability in the given parameter range (see Additional file 1, section 1).

**Figure 1:**
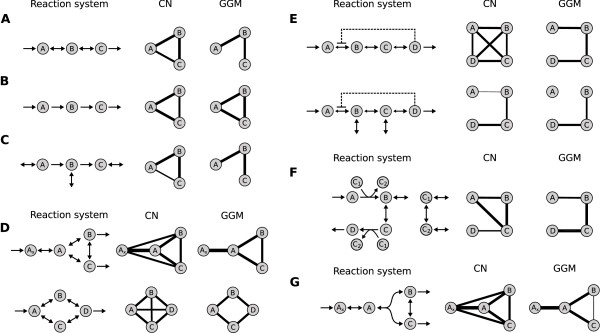
**Evaluation of correlation networks (CN) and Gaussian graphical models (GGM) on artificial systems**. Line widths represent relative edge weights in the respective networks (scaled to the strongest edges). **A: **Linear chain of three metabolites with reversible intermediate reactions. While the standard Pearson correlation network (CN) is fully connected, implying an overall high correlation of all metabolites, the GGM correctly discriminates between direct and indirect interactions. **B: **Linear chain with irreversible intermediate reactions. Neither CN nor GGM can distinguish direct from indirect effects, as metabolite A equally determines the levels of both B and C. **C: **Linear chain with irreversible reactions and input/output reactions for each metabolite. Although the edge weights for both CN and GGM are generally lower, the GGM now correctly predicts the network topology. **D: **Branched-chain first-order networks are correctly reconstructed by the GGM. **E: **End-product inhibition modules. When modeled as an open system, *A *is decoupled from the other metabolites and reconstruction fails at this point. Dashed lines mark enzyme inhibition interactions, larger arrows to the right indicate faster forward than backward reactions. **F: **Cofactor-driven network resembling the first three reactions from the glycolysis pathway. A correlation network fails to predict the correct pathway relationships. **G: **Non-linear system with a bi-molecular reaction. The GGM predicts only a only weak interaction between B and C. This is due to counterantagonistic processes of isomerization and substrate participation in the same reaction.

The first network we analyzed consists of a linear chain of three metabolites with different variants of reaction reversibility (Figure 1A-C). We observe high pairwise correlations for metabolites in mutual equilibrium due to reversible reactions (Figure 1A). This is in accordance with previous findings from [6], where correlation-generating mechanisms in metabolic reaction networks were identified. Furthermore, this simple example demonstrates how partial correlation coefficients in GGMs discriminate between directly and indirectly related metabolites. If only irreversible reactions are employed in the chain, neither regular correlation networks nor GGMs can distinguish between direct and indirect effects (Figure 1B). Species A is the only input metabolite in the system, and thus completely determines the levels of both B and C. This leads to generally high and non-distinguishable correlations between the three metabolites. However, if we introduce exchange reactions for all species, the GGM again correctly describes the network connectivity (Figure 1C). Such exchange mechanisms are likely to be present for most intracellular metabolites, which usually participate in multiple metabolic pathways (see e.g. KEGG PATHWAY online). Note that for this third case both regular and partial correlation values are notably lower than for the first two chain variants. In addition to linear chains, pathway modules consisting of branched topologies with first-order, reversible reactions are correctly reconstructed by our method (Figure 1D). An overview of the reconstruction accuracy of GGMs on various types of first-order networks with different variants of reaction reversibility can be found in Additional file 1, section 2.

Interestingly, for some reaction setups, the accuracy of the method improves drastically with an increasing amount of external noise. Specifically, if the metabolite transport towards a pathway is subject to higher fluctuations, the GGM edge weight difference between directly and indirectly connected metabolites becomes larger. For a detailed discussion of this finding we refer the reader to Additional file 1, section 3. The second question we addressed with artificial reaction networks was the influence of enzyme-catalyzed reactions on GGM estimation. Therefore we setup reaction chains with four metabolites incorporating reversible enzymatic reactions. Forward maximal reaction rates *V*_max _were set twice as fast as the backward reactions in order to ensure a directed mass flow. We found that the usage of Michaelis-Menten-type enzyme kinetics instead of mass-action kinetics does not alter our general findings. When forward reaction rates exceed backward reactions by far, the GGM discrimination quality is impaired. This is in line with the observation that purely irreversible reactions cannot be distinguished in the mass-action case (see above). Other specific parameters, like the Michaelis constant *K_M _*, did not affect GGM calculation (Additional file 1, section 4). Another important aspect of enzyme-catalyzed reactions are allosteric regulation mechanism, like end-product inhibition for instance, which constitutes a negative feedback from the end to the beginning of a pathway [22]. The reconstruction results differ depending on whether exchange reactions are included in the system for not (Figure 1E). If the inhibitory module represents a closed system (no external fluxes except for the first and last metabolite), the regulatory interaction does not in influence GGM calculation. The net metabolite turnover speed might be drastically affected, but the topological effects of this reaction chain on the correlation structure remain unchanged. In contrast, when exchange reactions are introduced (second example in Figure 1E), the inhibition decouples A from the other metabolites and the reconstruction fails for this metabolite. Detailed results for different strengths of the inhibitory interaction are presented in Additional file 1, section 5.

Next, we studied the influence of cofactor-driven reactions on the reconstruction. Cofactors are ubiquitous substances usually involved in the transfer of certain molecular moieties or redox potentials [23]. We investigated such cofactor-coupled reactions (a) because they introduce non-linearity in the simulated dynamical systems, and (b) because cofactors are usually involved in many reactions and thus generate network-wide metabolite dependencies. We set up a network resembling the first three reactions from the glycolysis pathway. It consists of four metabolites and two energy transfer-related cofactors, ATP and ADP, involved in two phosphorylation reactions [24]. Again the GGM precisely describes metabolite connectivity in the system, whereas a regular correlation graph leads to false interpretations of the network topology (Figure 1F). Cofactors were modeled with input and output reactions to the rest of the metabolic system in order to account for the above-mentioned participation of cofactors in various reactions of the system. Again, it makes a substantial difference whether such exchange reactions are included in the model or not. Since our toy model only represents a small part of a larger system, missing exchange reaction for cofactors would create a false mass conservation relation that compromises correlation calculation. Finally, we investigated the effects of rate laws with non-linear substrate dependencies in the absence of cofactors. Therefore we modeled a reversible, bimolecular split reaction with isomerization of the two substrates (Figure 1G). An example of such a reaction network can be found in the glycolysis pathway between *fructose-1,6-bisphosphate, glyceraldehyde-3-phosphate and dihydroxyacetone phosphate*. Our simulations demonstrate that again a regular Pearson correlation network cannot delineate direct from indirect relationships in the pathway. The GGM only detects a weak association between B and C. This is due to counterantagonistic processes in this reaction setup: isomerization and other reversible reactions generally induce positive correlations, whereas coparticipation as substrates in the same reaction induces negative correlations. Such effects of correlation-generating mechanisms which cancel each other out have been described before [6] and pose a problem to all reconstruction approaches which rely on linear dependencies.

The drawbacks of correlation-based methods discussed in this section, especially inhibitory mechanisms with exchange reactions and antagonistic mechanism, have to be kept in mind when attempting to reconstruct metabolic reactions from steady state data. For the present study, however, we assume the primarily linear lipid pathways not to contain such problematic reaction motifs.

### A GGM inferred from a large-scale population-based data set displays a sparse, modular and robust structure

**Figure 2:**
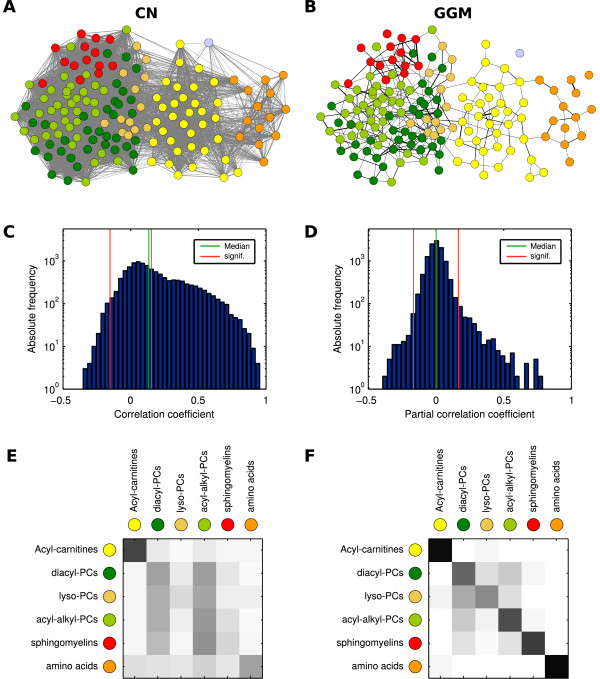
**Network properties of the correlation network (CN) and Gaussian graphical model (GGM) inferred from a targeted metabolomics population data set (1020 participants, 151 quantified metabolites)**. **A+B: **Graphical depiction of significantly positive edges in both networks, emphasizing local clustering structures. Each circle color represents a single metabolite class. **C+D: **Histograms of $\left(\begin{array}{l} 151\\ \;2 \end{array}\right)\;=\;11325$ pairwise correlation coefficients (i.e. edge weights) for both networks. Green lines indicate the median values, red lines denote a significance level of 0.01 with Bonferroni correction. The CN displays a general bias towards positive correlations throughout all metabolites. For the GGM, the median value lies around zero and we observe a shift towards significantly positive values. **E+F: **Modularity between metabolite classes measured as the relative out-degree from each class (rows) to all other classes (columns). The GGM (right) shows a clear separation of metabolite classes, with some overlaps for the different phospholipid species diacyl-PCs, lyso-PCs, acyl-alkyl-PCs and sphingomyelins. Values range from white (0.0 out-degree towards this class) to black (1.0). PCs = phosphatidylcholines.

In the following we estimated a Gaussian graphical model using targeted metabolomics data from the German population study KORA [18] ("Kooperative Gesundheitsforschung in der Region Augsburg"). We used a subset of the data set previously evaluated in a genome-wide association study [19], containing 1020 targeted metabolomics fasting blood serum measurements with 151 quantified metabolites. The metabolite panel includes acyl-carnitines, four classes of phospholipid species, amino acids and hexoses (see Methods). Both regular Pearson correlation coefficients and partial correlation coefficients (inducing the GGM) were calculated on the logarithmized metabolite concentrations. All edges corresponding to correlation values significantly different from zero now induce the networks displayed in Figure 2A+B. In order to exclude correlation effects generated by genetic variation in the study cohort, we investigated the in influence of SNP allele data from [19] on the GGM calculation. We found genetic effects to be neglectable (see Additional file 2), indicating that GGMs capture intrinsic biochemical properties of the system.

Pearson correlation coefficients show a strong bias towards positive values in our data set (Figure 2C); a typical feature of high-throughput data sets, also observed e.g. in microrarray expression data, which can be attributed to unspecific or indirect interactions [10]. We obtain 5479 correlation values significantly different from zero with $\tilde{\alpha }=8.83.10^{-7}$ (α = 0.01 after Bonferroni correction), yielding an absolute significance correlation cutoff value of 0.1619 (see Methods). In contrast, the GGM shows a much sparser structure with 417 significant partial correlations after Bonferroni correction (Figure 2D). Most values center around a partial correlation coefficient of zero, whereas we observe a clear shift towards positive significant values. Note that negative partial correlations provide particular information that will be discussed later in this manuscript.

The GGM displays a modular structure with respect to the seven metabolite classes in our panel, while the class separation in the correlation network appears rather blurry (Figure 2E+F). We observe a clear separation of the amino acids and acyl-carnitines from all other classes. The four groups of phospholipids (diacyl-PCs, lyso-PCs, acyl-alkyl-PCs, and sphingomyelins) still showed locally clustered structures, but are strongly interwoven in the network. This is probably an effect of the dependence of all phospholipids on a similar fatty acid pool and, subsequently, the biosynthesis pathway acting on this substrate pool. In order to get an objective quantification of this observation, we calculated the group-based modularity *Q *on all significantly positive GGM edges according to [25] (see Methods). The same measure was calculated for 10^5 ^randomized GGM networks (random edge rewiring). For the original GGM we obtain a modularity of *Q *= 0.488, and the random networks yield *Q *= 0.118 ± 0.016, resulting in a highly significant *z*-score of *z *= 23.49. Furthermore, the modularity value induced by using the metabolite classes was compared to a partitioning optimized by simulated annealing. The optimized modularity is only slightly higher with *Q *= 0.557 and the resulting partitioning is very similar to the metabolite classes (see Additional file 3). Performing the modularity analysis with the full, weighted partial correlation matrix produces equivalent results (also shown in S3).

An important question for a multivariate statistical measure such as partial correlations is the robustness with respect to changes in the underlying data set. Furthermore, the dependence of the measure on the size of the data set needs to be addressed. To answer these questions, we performed two types of perturbations of our data set. First, we applied sample bootstrapping with 1000 repetitions and compared the resulting partial correlations to the original data set (Additional file 4, Figure S1). We observe small mean differences with low standard deviation (0.03 ± 8. 2 · 10^-4^). This indicates that for a large data set with *n *= 1020 samples, GGMs are robust against the choice of samples. We assume that each distinct metabolic state in the cohort is captured by a bootstrap sample, and thus all information required to calculate the GGM is contained. In addition to the bootstrap analysis, we estimated partial correlations for continuously decreasing sample sizes (Additional file 4, Figure S2). For each data set size we randomly picked samples from the original data set and repeated the procedure 100 times. The analysis shows that the GGM is stable even under decrease of the sample number. For instance, for a data set containing only around half of the original samples (*n *= 530) we get a partial correlation difference of 0.03 ± 6.9 10^-4^. Only when the number of samples gets close to the number of variables (*m *= 151) the correlation matrix becomes ill-conditioned and strong differences from the original partial correlations occur. These problems of smaller metabolomics studies could be dealt with by regularization approaches or the usage of low-order partial correlation [26]. Taken together, our results demonstrate that the analyzed metabolomics data set is sufficient to robustly elucidate relationships between the measured metabolites.

### Strong GGM edges represent known metabolic pathway interactions

The next step in our analysis was the manual investigation of metabolite pairs displaying strong partial correlation coefficients. Remarkably, we are able to provide pathway explanations for most metabolite pairs in the top 20 positive partial correlations (Table 1). In the following, we will specifically discuss interesting, high-scoring metabolite pairs along with their responsible enzymes in the metabolic pathways.

**Table 1:** Top 20 positive GGM edge weights (i.e. partial correlation coefficients, PCC) in our data set along with proposed metabolic pathway explanations

Metabolite 1	Metabolite 2	PCC	Comment
Val	xLeu	0.821	Branched-chain amino acids
SM C18:0	SM C18:1	0.767	SCD/SCD5 desaturation
SM C16:1	SM C18:1	0.765	ELOVL6
PC ae C34:2	PC ae C36:3	0.752	2 reaction steps
SM (OH) C22:1	SM (OH) C22:2	0.743	sphingolipid-specific desaturation?
PC aa C34:2	PC aa C36:2	0.735	ELOVL1/ELOVL6 elongation
C10:0-carn	C8:0-carn	0.735	*β*-oxidation step
lysoPC a C16:0	lysoPC a C18:0	0.731	ELOVL6 elongation
PC aa C38:6	PC aa C40:6	0.709	ACOX1/3 + various ELOVLs
SM (OH) C14:1	SM (OH) C16:1	0.686	sphingolipid-specific elongation?
PC aa C36:4	PC aa C38:4	0.672	ACOX1/3 + various ELOVLs
PC aa C32:1	lysoPC a C16:1	0.661	C16:0/C16:1 phospholipid association
PC aa C38:5	PC aa C40:5	0.653	various ELOVLs
PC ae C34:3	PC ae C36:5	0.607	at least 3 reaction steps
PC aa C36:5	PC aa C38:5	0.596	ACOX1/3 + various ELOVLs
SM C24:0	SM C24:1	0.577	sphingolipid-specific desaturation?
PC ae C32:1	PC ae C32:2	0.574	SCD/SCD5 desaturation
SM (OH) C22:2	SM C24:1	0.567	possible elongation intermediate
C18:1-carn	C18:2-carn	0.561	*β*-oxidation intermediate

Most metabolite pairs can be directly linked to reactions in the fatty acid biosynthesis pathway, the *β*-oxidation pathway or amino acid-associated pathways.

The highest partial correlation in the data set with ζ = 0.821 is found for the two branched-chain amino acids Valine and xLeucine, where the latter compound represents both Leucine and Isoleucine (which have equal masses and are not distinguishable by the present method). The three metabolites are in close proximity in the metabolic network concerning their biosynthesis and degradation pathways. Further related amino acid pairs that display significant partial correlations are Histidine and Glutamine (ζ = 0.383), Glycine and Serine (ζ = 0.326) as well as Threonine and Methionine (ζ = 0.298).

**Figure 3:**
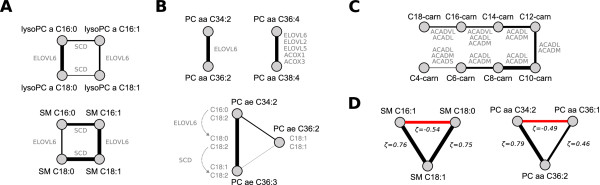
**Biochemical subnetworks identified by the GGM**. Line widths correspond to partial correlation coefficients. **A: **Elongation and desaturation signatures, most likely mediated by ELOVL6 and SCD, for C16 and C18 fatty acids incorporated in lyso-PCs and sphingomyelins. **B: **Top: Diacyl-phosphatidylcholine (PC aa) species with elongation and peroxisomal *β*-oxidation associations. Several combinatorial variants of side chain compositions are possible for C36:4 and C38:4, and thus different enzymes could mediate this connection. Bottom: Alkyl-acyl-phosphatidylcholines (PC ae) with supposedly distinct side chain composition, giving rise to a low association with a directly connected species (C36:2). **C: **Recovered *β*-oxidation pathway from C18 down to C4. Four enzymes with overlapping substrate specificities catalyze the rate-limiting reactions of this pathway. **D: **Two high-scoring triads, where metabolite pairs with a pathway distance of two constitute strong partial correlations. This feature of partial correlations aids in the reconstruction of the network topology beyond the direct neighborhood of each metabolite.

Clear-cut signatures of the desaturation and elongation of long chain fatty acids can be seen for various sphingomyelins and lyso-PCs (Figure 3A). For example, SM C18:0 and SM C18:1 strongly associate with ζ = 0.767, most probably representing the initial Δ9 desaturation step of the polyunsaturated fatty acid biosynthesis pathway from C18:0 to C18:1-Δ9 by SCD (*Steaoryl-CoA desaturase*). The similarly high partial correlation between SM C16:1 and SM C18:1 (ζ = 0.765) as well as lysoPC a C16:1 and lysoPC a C18:1 (ζ = 0.315) can be attributed to the ELOVL6-dependent elongation from C16:1-Δ 9 to C18:1-Δ 11. Interestingly, this reaction is not contained in the public reaction databases but has been previously described by [27].

We identify a variety of strong GGM edges between diacyl-PC (lecithins, PC aa) and acyl-alkyl-PC (plasmalogens, PC ae) metabolite pairs (Figure 3B). For instance, PC aa C34:2 and PC aa C36:2 associate strongly with ζ = 0.735, and PC aa C36:4 and PC aa C38:4 show a partial correlation of ζ = 0.672. While the first pair can be precisely explained by an elongation from C16:0 to C18:0 by ELOVL6, different combinatorial variants come into play for the PC aa C36:4/PC aa C38:4 pair. Our mass-spectrometry technique only measures *brutto *compositions, that is the bulk side chain carbon content and total degree of desaturation. Depending on the exact composition of both fatty acid residues in the respective lipids, this association could be caused by long-chain elongations (C14 to C16 and C16 to C18 through fatty acid synthase and ELOVL6, respectively), by very-long-chain elongations (C22:4 to C24:4 through ELOVL2 or ELOVL5) and even by peroxisomal *β *-oxidation of fatty acids (through ACOX1 or ACOX3). An interesting situation arises for the phospholipids PC ae C34:2, PC ae C36:3 and PC ae C36:2. From its brutto formula the latter species could represent an intermediate step between the other two metabolites. However, it associates poorly with both other phospholipids, which in turn display a strong partial correlation (ζ = 0.752). This finding can be explained by distinct fatty acid side chain compositions, showing differential incorporation of C18:0, C18:1 and C18:2 (Figure 3B, bottom).

For the acyl-carnitine group we observe a remarkably high partial correlation of ζ = 0.735 for C8-carn and C10-carn and further acyl-carnitine pairs with a carbon atom difference of two (Figure 3C). These associations can be attributed to the *β*-oxidation pathway, i.e. the catabolic breakdown of fatty acids in the mitochondria [23]. During this degradation process, C_2 _units are continuously split off from the shrinking fatty acid chain. Four *acyl-CoA dehydrogenases*, ACADS, ACADM and ACADL, ACADVL, catalyze the rate limiting reactions of *β*-oxidation for different fatty acid chain lengths [28,29]. Our interpretation of acyl-carnitine correlations as signatures of mitochondrial *β*-oxidation is in accordance with [19], where we identified associations between C8+C10, C12 and C4 with genetic variation in the ACADM, ACADL and ACADS loci, respectively.

We observe several associations that were not directly attributable to enzymatic interactions in the fatty acid biosynthesis or degradation pathways. For instance, lysoPC a 18:1 and lysoPC a 18:2 share a strong GGM edge (ζ = 0.543) although the Δ12-desaturation step from oleic acid to linoleic acid is known to be missing in humans [30]. This missing reaction gives rise to the *essentiality *of fatty acids in the *ω*-6 unsaturated fatty acid pathway. A functional explanation could be a systemic equilibrium between the two fatty acids or remodeling processes specific for the lyso-PC metabolite class. Further examples are high partial correlations between the hydroxy sphingomyelins SM (OH) C22:1 and SM (OH) C22:2 (ζ = 0.743) as well as the sphingomyelins SM C24:0 and SM C24:1 (ζ = 0.577). To the best of our knowledge, there is no evidence for such fatty acid desaturation reactions in humans. The detected associations might therefore represent novel pathway interactions recovered by the Gaussian graphical model.

Negative values play a particular role in the interpretation of partial correlations coefficients. On the one hand, they obviously occur whenever regular negative correlations are involved. Mechanisms giving rise to negative correlations are, for example, coparticipation in the same reaction (cf. Figure 1E), mass conservation relations [6] or opposing regulatory effects. It is to be noted, however, that negative correlations are rare in our specific metabolomics data set (cf. Figure 2C). On the other hand, due to the mathematical properties of partial correlation coefficients negative partial correlation coefficients occur whenever two metabolites *A *and *B *have a strong correlation with a third metabolite *C*, but do not share a high correlation value with each other. Two examples from our data set are shown in Figure 3D. First, SM C18:0 is negatively partially correlated with SM C16:1, and both of these in turn are highly positively partially correlated with SM C18:1. The fatty acids C16:1 and C18:0 have no direct connection in the pathway, causing the strong negative partial correlation value. A similar situation can be found for three diacyl-PCs: PC aa C34:2 and PC aa C36:1 show a high partial correlation with PC aa C36:2, but a negative partial correlation with each other. Again, there is no possible direct reaction from a C34:2 lipid species to a C36:1 species. Not all metabolite triads in the network show such a one-negative/two-positive motif. But if present, they provide another step in the reconstruction of metabolic pathways (beyond the direct neighborhood of each metabolite) by detecting metabolites which are exactly two steps apart.

### Partial correlation coefficients discriminate between directly and indirectly connected metabolites in a literature-curated fatty acid pathway model

**Figure 4:**
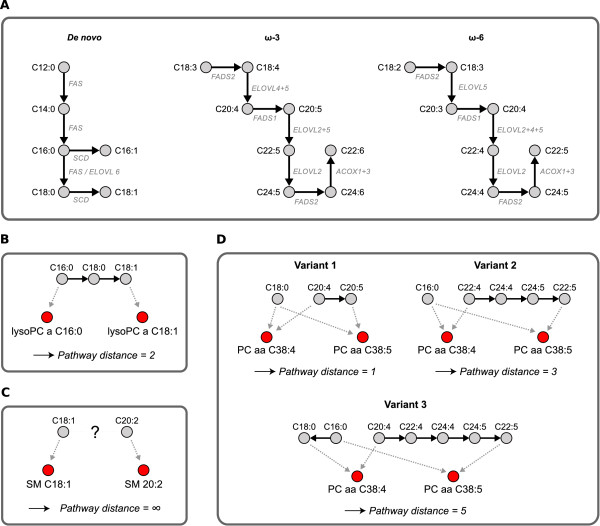
**Fatty acid biosynthesis model and pathway distance calculation method**. **A: ***De novo *synthesis of fatty acids with initial SCD-dependent desaturations (left), and the *ω*-3 and *ω*-6 poly-unsaturated fatty acid pathways (middle and right). Note that we omitted the specific positions of each double-bond since the mass-spectrometry technique in our study does not resolve positional information. **B: **Exemplary distance calculation on two lyso-PCs. We projected lipid side chain compositions onto the respective fatty-acid biosynthesis reactions. Reaction reversibility is not taken into account in our calculation, i.e. distances are always symmetric. **C: **If no known pathway connection between two fatty acids exists, we assign a formal distance of infinity. **D: **For phospholipids that contain two fatty acid residues we need to take into account all combinatorial variants. We here show three variants for the connection between PC aa C38:4 and PC aa C38:5. In these examples, PC aa C38:4 could either consist of C18:0+C20:4 or C16:0+C22:4, while PC aa C38:5 could be C18:0+C20:5 or C16:0+C22:5. The shortest possible distance, one in this case, will be used for further calculations.

The analyses from the previous section strengthened our conception that a GGM inferred from blood serum metabolomics data represents true metabolite associations. To systematically assess how GGM edges and pathway proximity between our lipid metabolites are related, we generated a literature-based model of fatty acid biosynthesis (Figure 4A). This model includes reactions from the public databases BiGG (H. sapiens Recon 1) [7], the Edinburgh Human Metabolic Network [31] and KEGG PATHWAY [29]. We then mapped the partial correlation coefficients from the KORA data set onto the minimal number of reaction steps between each pair of metabolites (*pathway distance*). Since our metabolite panel contains fatty-acid based lipids, we project the respective lipid compositions onto the fatty acid biosynthesis pathway (Figure 4B-D). For the analysis of acyl-carnitines we implemented a model of the *β*-oxidation pathway, consisting of a linear chain of C2 degradation steps (C10→C8→C6 etc.).

**Figure 5:**
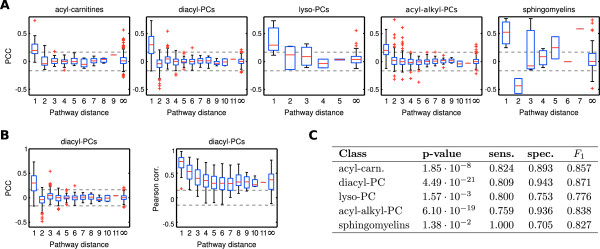
**Systematic evaluation of partial correlation coefficients versus pathway distances**. Dashed lines in A and B indicate a significance level of 0.01 with Bonferroni correction. **A: **Pathway distances from our consensus model against partial correlation coefficients for the five lipid-based metabolite classes in our data set. We observe an enrichment of significant partial correlations for a pathway distance of one, which rapidly drops for an increasing number of pathway steps. **B: **Comparison of partial correlation coefficients and Pearson correlation coefficients. Pearson correlation coefficients are generally high, independent of the actual pathway distance, indicating for systemic coregulation effects throughout the lipid metabolism. **C: **Wilcoxon rank sum test p-values between the partial correlation distributions of directly and indirectly connected pairs, and sensitivity/specificity/*F_1 _*values measuring the discriminatory power to distinguish direct from indirect pairs.

We observe a strong tendency towards significantly positive partial correlations for a pathway distance of one, i.e. directly connected metabolite pairs, for all five metabolite classes (Figure 5A). In total, 86 out of 130 partial correlations (66%) for a pathway distance of one are significantly positive. For instance, for the lyso-PC class (Figure 5A) nearly all partial correlation coefficients for a pathway distance of one are above significance level, whereas most values for a distance of two or larger remain insignificant. Some outliers from this observation, however, require closer inspection: First, for some metabolite classes we observe negative partial correlation values for metabolite pairs that are exactly two steps apart in the metabolic pathway: 10 of 73 partial correlations in the diacyl-PC class and 2 of 2 partial correlations in the sphingomyelin class are significantly negative for a distance of two. These negative values are effects of the coregulated metabolite triads described previously in this text. Second, we find 91 of 932 (~9:8%) unconnected metabolite pairs (pathway distance = ∞) with a partial correlation above significance level. These pairs represent potentially novel pathway predictions, missing interactions in the model or effects upstream of the metabolic network like enzyme coregulation.

A direct comparison of both partial and Pearson correlation coefficients for the diacyl-phosphatidylcholine class is shown in Figure 5B. As described earlier in this manuscript, we observe a general over-abundance of significant Pearson correlations independent of the actual pathway distance. Even for the metabolites without a known pathway connection, 1394 of a total of 1569 Pearson correlations are significant (88.85%, over all classes), in contrast to 131 out of 1569 for the partial correlations (8.35%).

The significantly different correlation value distributions between directly and indirectly linked metabolites (Figure 5A+B) barely provide a good quantification of the actual discrimination accuracy of this feature. Therefore we assessed the discriminative power of partial correlations to tell apart direct from indirect interactions by means of *sensitivity *and *specificity*. The sensitivity evaluates which fraction of directly connected metabolites in the pathway are recovered by significant GGM edges, whereas the specificity states how many of the significant edges actually represent a direct connection. A commonly used trade off measure between sensitivity and specificity is the *F*_1 _score, which is defined as the harmonic mean of both quantities [32] (see Methods). Figure 5C lists sensitivity, specific city and F_1 _for all 5 metabolite classes along with an evaluation of partial correlation distribution differences between directly and indirectly linked metabolites (determined by Wilcoxon's ranksum test). *F*_1 _values over 0.75 and significant p-values for the ranksum test indicate a strong discrimination effect of partial correlation coefficients concerning direct vs. indirect pathway interactions. Possible reasons for non-perfect sensitivity and specific city values will be discussed in detail at the end of this text.

### Low-order partial correlations

The data set from our present study contained enough samples to calculate full-order partial correlations, that is to calculate pairwise correlations conditioned against all other *n*-2 metabolites. However, previous studies demonstrated that low-order partial correlation approaches can already be sufficient to elucidate direct interactions [12,16]. In order to assess how these measures perform in comparison to the full-order GGM, we calculated first-, second- and third order partial correlations using the approach developed by [12] for both computer-simulated networks and the metabolomics data (Additional file 5). The toy systems reveal clear cases where low-order approaches fail, for instance in the *diamond *motif displayed in Figure 1D. Surprisingly, however, especially first-order partial correlations worked remarkably well in discriminating direct from indirect interactions in the real data (F_1 _values close to those displayed in Figure 5C). This result provides two valuable pieces of information. First, low-order partial correlation approaches, which require much less samples to obtain stable estimates, appear to be a suitable alternative to GGMs for the metabolite panel used in this study. Second, the high relative scoring of first-order partial correlations provides insights into the correlation structures in the data set. In particular, this result indicates that the underlying metabolic pathways are primarily composed of acyclic, linear chains, which fits well to the fatty acid pathways dominating our measured lipid species.

## Conclusions

In this paper we addressed the reconstruction of metabolic pathway reactions from high-throughput targeted metabolomics measurements. Previous reconstruction approaches employed pairwise association measures, primarily standard Pearson correlation coefficients, to infer network topology information from metabolite profiles [5,6,8,33]. We here demonstrated the usefulness of Gaussian graphical models and their ability to distinguish direct from indirect associations by estimating the *conditional *dependence between variables. GGMs are based on partial correlation coefficients, that is the Pearson correlation between two metabolites corrected for the correlations with all other metabolites.

From computer simulations of metabolic reaction networks we deduced a set important aspects to be considered when interpreting partial correlation coefficients in reaction systems: (a) Metabolites in equilibrium due to reversible reactions can readily be recovered, whereas irreversible reactions pose a substantial problem to association-based reconstruction attempts (in concordance with [6]). (b) Input and output reactions for intermediate metabolites, however, improve the reconstruction accuracy. Such exchange reactions are likely to be present for most naturally occurring metabolites due to highly interconnected metabolic pathways. (c) With an increasing amount of fluctuations on the input reaction, the partial correlation difference between direct and indirect interactions increases for certain network topologies (e.g. for the irreversible linear metabolite chains). This indicates that a high heterogeneity of metabolic states in a population data set like the KORA cohort might be beneficial rather than problematic for our approach. (d) Metabolite connectivity in cofactor-driven networks can be accurately reconstructed. The presence of exchange reactions for cofactors, as they are likely to be present in real systems, has substantial impact on the reconstruction quality. The connectivity of the cofactors themselves, however, remains spurious. (e) Saturation effects in enzyme-catalyzed reactions do not pose a problem for the reconstruction process. However, inhibitory influences in metabolic modules that include exchange reactions might decouple certain metabolites and lead to false negative results. (f) Non-linear rate laws and antagonistic, correlation-generating mechanisms might impair reconstruction quality.

In the next step we inferred both a GGM and a regular correlation network from a large-scale metabolomics data set with 1020 strictly standardized samples from overnight fasting individuals measured by state-of-the art metabolomics technologies [19]. We investigated the influence of the 15 genome-wide-significant SNPs from this study on our GGM and demonstrated that genetic variation in the general population is neglectable for partial correlation calculation. We found that the GGM displays a much sparser structure than regular correlation networks. Only around 400 partial correlation values were above significance level (~3.6%), whereas half of all Pearson correlation values were significant after Bonferroni correction. This depicted the nature of partial correlation coefficients to neglect indirect associations between distantly related metabolites. We detected a strongly modular structure in the GGM with respect to the different metabolite classes, except for the four types of phospholipids which appear slightly interwoven. This provides a unique picture of the separation of metabolic pathways (synthesis, degradation and amino acid metabolism), but also the interaction between different lipid classes dependent on a single intracellular fatty acid pool. Finally, GGMs were stable with respect to both choice and number of samples in the data set. Even a smaller data set with only a few hundred samples would have been sufficient to achieve the results from this study. The estimation of GGMs for data sets with less samples than metabolites is possible [26], but notable deviations from the true partial correlation coefficient shave to be expected.

Manual investigation of high-scoring substructures in the GGM revealed groups of metabolites that could be directly attributed to reaction steps from the human fatty acid biosynthesis and degradation pathways. We detected effects of ELOVL-mediated elongations and FADS-mediated desaturations of fatty acids as well as signatures of the catabolic *β*-oxidation pathway. For instance, our method successfully recovered a direct elongation from C16:1 to C18:1, which has been experimentally shown by [27] but is not present in the public reaction databases. Furthermore, we identified highly negative partial correlations as an indication for a pathway distance of two, serving as a further hint in the reconstruction of metabolic network topology. In order to systematically evaluate whether high partial correlations represent direct interactions, we generated a consensus model of fatty acid biosynthesis reactions from three publically available reaction databases. By mapping partial correlation coefficients to the number of reaction steps between two metabolites we observed a statistically significant enrichment of high values for a pathway distance of one. We calculated a high accuracy for partial correlations to discriminate between directly and indirectly associated metabolites, as measured by sensitivity, specificity and the *F*_1 _measure. Interestingly, we could show that the discrimination quality of low-order partial correlations [12], especially the first-order variants, is close to the full-order GGM. Even though this might be a feature specific to the metabolite panel used in this study, low-order partial correlations represent a suitable alternative especially for studies with only few samples. If more samples than variables are available, however, we recommend GGMs as an unbiased approach conditioning against as many parameters as possible.

Taken together, our results demonstrate that GGMs inferred from metabolomics measurements in blood plasma samples reveal strong signatures of intracellular and even inner-mitochondrial processes. Previous studies on blood plasma samples detected similar relationships with cellular processes based on genetic associations [19] and case/control drug trials [34]. In this work we could now show that metabolite profiles alone are sufficient to capture the dynamics of metabolic pathways.

However, GGMs can never provide a perfect reconstruction of the underlying system. There are several factors that lead to the absence of high partial correlations between interacting metabolites, that is false negative edges in the GGM: (a) Counterantagonistic correlation-generating processes and bimolecular reactions (see above) might lead to the elimination of pairwise association; cf. [6]. (b) The respective enzyme might not be active in the current metabolic state, or its effects on the respective metabolite pools are neglectable. (c) Contrary to our general finding that even blood plasma metabolites carry strong signatures of metabolic pathways, the signal might be diminished for certain types of metabolites. Furthermore, the actual origins of blood plasma metabolites, e.g. in terms of measured cell types or causal tissue activity, still remain to be unraveled. The above-mentioned mechanisms are possible explanations for the non-perfect sensitivity values observed in Figure 5C. False positive GGM edges, on the other hand, provide interesting new metabolic pathway hypothesis. The presence of strong partial correlations in the absence of known metabolic connections could point out missing pathway information or regulatory effects not captured in a simple stoichiometric representation of the pathway.

Conclusively, this study presented Gaussian graphical models as a valuable tool for the recovery of biochemical reactions from high-throughput targeted metabolomics data. The present work could be extended by comparing high partial correlation coefficients with enzyme activity or expression data, or by the experimental validation of promising interaction candidates. We suggest using GGMs as a standard tool of investigation in future metabolomics studies, utilizing the upcoming wealth of metabolic profiling data to form a more comprehensive picture of cellular metabolism.

## Methods

### In silico simulation of artificial reaction networks

Let *x *= (*x*_1_,..., *x*_r_) be a vector of metabolite concentrations and *S ∈ *ℤ^*m*×*r *^the stoichiometry matrix of a dynamical system with *m *metabolites and *r *reactions. Each column in *S *represents the compound stoichiometry of a single reaction, with negative values for the educts of a reaction and positive values for its products (cf. [35]). Furthermore, we define an *educt stoichiometry matrix S*^e^, which only contains the negative values from *S*. The reaction rate laws *v *can be written as *v*(*x*, *k*) = diag(*k*)*c*(*x*), where *k *:= (*k*_1_,...,*k*_r_) represents a vector of elementary rate constants and $c_{j}\left(x\right):=\prod_{i=1}^{m}\;x_{i}^{-s_{ij}^{e}},\;j=1,...,r$ contains the products of substrate concentrations according to the law of mass action[36]. For example, for the reaction *x*_1 _+ *x*_2 _→ *x*_3 _we obtain *c *= *x*_1_*x*_2_, and 2*x*_1 _+ 3*x*_2 _→ 2*x*_3 _yields *c *= *x*_1_^2^*x*_2_^3^. For enzyme-catalyzed reactions *i*, the corresponding entries in *v *are formulated using reversible Michaelis-Menten-type kinetics [37,38] instead of the mass-action term above:

(1) $$v_{i}=\frac{\frac{V_{\max}^{+}}{K_{M}^{\text{s}}}\cdot [S]-\frac{V_{\max}^{-}}{K_{M}^{p}}\cdot [P]}{1+\frac{\left[S\right]}{K_{M}^{\text{s}}}+\frac{\left[P\right]}{K_{M}^{p}}}$$

Where $V_{\text{max}}^{+}$ and $V_{\text{max}}^{-}$ are the product and substrate formation constants, respectively, $K_{M}^{s}$ and $K_{M}^{p}$ represent the Michaelis constants for substrate and product, [*S*] represents the substrate concentration and [*P*] represents the product concentration. Note that we omitted reaction-specific parameter indices for simplicity here. Allosteric regulation was modeled using a mixed inhibition mechanism, which extends the rate law from equation (1) as follows:

$$v_{i}=\frac{\frac{V_{\max}^{+}}{K_{M}^{\text{s}}}\cdot \left[S\right]-\frac{V_{\max}^{-}}{K_{M}^{p}}\cdot \left[P\right]}{1+\frac{\left[I\right]}{K_{i}}+\left(\frac{\left[S\right]}{K_{M}^{\text{s}}}+\frac{\left[P\right]}{K_{M}^{p}}\right)\left(1+\frac{\left[I\right]}{K_{ii}}\right)}$$

with [*I*] being the inhibitor concentration, *K_i _*the binding rate of the inhibitor to the enzyme and *K_ii _*the binding rate of the inhibitor to the substrate-enzyme (or product-enzyme) complex. In a simple mixed (*non-competitive*) inhibition scenario, we assume *K_i _= K_ii_*.

The ordinary differential equations describing the temporal evolution of the system are now given as

(2) $$\frac{\text{d}x}{\text{d}t}=S\cdot v\left(x,\;k\right)$$

To introduce variability each parameter is subject to fluctuations according to a log-normal distribution with mean 1 and changing variances: $k_{i}\sim \text{LogN}\left(1,\;\sigma_{i}^{2}\right)$. Finally, for fixed *S *and *k*, Pearson and partial correlations (see below) are calculated by drawing the vector *k *multiple times from the parameter distribution, calculating the corresponding metabolite steady state concentrations and logarithmizing the obtained values. If the system contains only zeroth-order and first-order reactions (i.e. input reactions and reactions with only one substrate), the steady state concentrations for a given *k *can be readily computed by equating (2) to zero and solving for *c *using linear algebra techniques. On the other hand, if higher order reactions are present, the ODEs are integrated numerically and simulated until equilibrium to get corresponding steady states. For this purpose, a variable-order solver for stiff differential equations (ode15s) from MATLAB was used [39]. The presence of a unique, single positive steady state was shown for each network individually using the ERNEST toolbox [21], or by empirical evaluation (parameter and initial value sampling). For a detailed analysis we refer the reader to Additional file 1, section 1.

### Computation of correlation network and Gaussian graphical model

Let *X *= (*x_kl_*) be the ℝ^*n*×*m *^matrix of logarithmized metabolite concentrations (either measured data samples or computer-simulated steady states), where *n *is the number of samples, and *m *again represents the number of metabolites. Then the standard Pearson product-moment correlation coefficients *P *= (*ρ*_*ij*_) between metabolites are calculated as

$$\rho_{ij}=\frac{\sum_{k=1}^{n}\left(x_{ki}-{\bar{x}}_{i}\right)\left(x_{kj}-{\bar{x}}_{j}\right)}{\sqrt{\sum_{k=1}^{n}{\left(x_{ki}-{\bar{x}}_{i}\right)}^{2}}\;\cdot \;\sqrt{\sum_{k=1}^{n}{\left(x_{kj}-{\bar{x}}_{j}\right)}^{2}}}$$

where ${\bar{x}}_{i}$ represents the mean value of metabolite *i*. Since we use a Gaussian graphical model, the conditional distributions are also Gaussian. Their width and the corresponding partial correlation coefficients can be calculated as

$Z=\left(\zeta_{ij}\right)=-\omega_{ij}/\sqrt{\omega_{ii}\omega_{jj}}$ with $\left(\omega_{ij}\right)=P^{-1}$

A partial correlation value *ζ_ij _*denotes the pairwise correlation of metabolites *i *and *j *corrected for the effects of all remaining metabolites. Since our study design contains more samples than measured variables, the correlation matrix has full rank and its inverse can be straightforwardly determined. First-,second-, and third-order partial correlations were calculated using the software published in [12]. To assess the significance of partial correlations, p-values *p*(*ζ_ij_*) were calculated using Fisher's *z*-transform [40]:

(3) $$\begin{array}{c} z\left(\zeta_{ij}\right)=\frac{1}{2}\ln\left(\frac{1+\zeta_{ij}}{1-\zeta_{ij}}\right)\\ p\left(\zeta_{ij}\right)=\left(1-\phi \left(\sqrt{n-\left(m-2\right)-3}\cdot z\left(\zeta_{ij}\right)\right)\right)\cdot 2 \end{array}$$

where *ϕ *stands for the cumulative distribution function of the standard normal distribution. In order to account for multiple testing, Bonferroni correction was applied to obtain an estimate of the significance level. Note that Bonferroni correction is the most conservative approach for multiple testing; it assumes independence of all tested values, which is certainly not the case for partial correlation coefficients. Based on a nominal significance level of α = 0.01, we retrieve an adjusted level of $\tilde{\alpha }=0.01/11325=8.83\cdot 10^{-7}$ after Bonferroni correction. Solving equation (3) for *ζ_ij _*yields a minimum absolute partial correlation coefficient of 0.1619 for the given significance level. That is, all partial correlations smaller than -0.1619 or larger than 0.1619 are considered significant.

Bootstrapping was performed by randomly drawing 1020 samples with replacement from the original data set. For the second stability analysis, the investigation of different data set sizes, the respective number of samples was randomly drawn from the original data set. The whole procedure was repeated 100 times to get a stable estimate of the deviation.

### Network modularity calculation

We define the adjacency matrix *ξ_ij _*of a new unweighted, undirected graph induced by all significantly positive partial correlations in *ζ_ij_*:

$$\xi_{ij}:=\{\begin{array}{ll} 1, & \text{if}\;\zeta_{ij}\geq \tilde{\alpha }\\ 0, & \text{else} \end{array}$$

where $\tilde{\alpha }$ represents the significance level after multiple testing correction. Now let (*V*_1_,...,*V*_6_) be the partitioning of the metabolites into the six metabolite classes: acyl-carnitines, diacyl-PCs, lyso-PCs, acyl-alkyl-PCs, sphingomyelins and amino acids (the hexose is left out as only a single metabolite belongs to that class). We calculated the *relative out-degree R_ij _*∈ ℝ^6×6 ^from each class to the other classes, (i.e. the proportion of its edges each class shares with the other classes) as:

$$R_{ij}\;:=\;\frac{A\left(V_{i},V_{j}\right)}{A\left(V_{i},V\right)}$$

where $A\left(V^{\prime },\;V"\right)\;\;=\;\;\sum_{i\in V',j\in V"}\xi_{ij}$ represents the total number of edges between *V*' and *V*", and *V *= U*V_i _*contains all metabolites in the network. The total network modularity *Q *of the network can be quantified according to [41] as:

(4) $$Q:=\sum_{i=1}^{6}\left[\frac{A\left(V_{i},V_{i}\right)}{A\left(V,V\right)}-{\left(\frac{A\left(V_{i},V\right)}{A\left(V,V\right)}\right)}^{2}\right]$$

Intuitively, this measure compares the within-class edges with the edges to the rest of the network. The more edges there are within each class in comparison to the other classes, the higher *Q *will be. Note that equation (4) can be applied to both weighted and unweighted graphs. To assess the significance of the observed value, we performed graph randomization by edge rewiring [42,43] and subsequent calculation of *Q*. During the rewiring process we randomly pick two edges from the network and exchange the target nodes of each edge. In order to achieve sufficient randomization, this operation is repeated 5 · *e *times, where *e *represents the number of edges in the graph. To perform edge reshuffling on weighted graphs, we decided on a neighbor-preserving variant as described in [44].

### Study cohort and metabolite panel

KORA (Kooperative Gesundheitsforschung in der Region Augsburg) is a research platform in southern Germany with a primary focus on cardiovascular diseases, Diabetes mellitus type 2, and genetic epidemiology [18]. Fasting serum concentrations from *n *= 1020 individuals in the KORA F4 were determined by electrospray ionization tandem mass spectrometry (ESI-MS/MS) using the *Biocrates AbsoluteIDQ™*targeted metabolomics kit technology. These samples represent a subset of the data set previously evaluated in a genome-wide association study in [19].

A total of *m *= 151 metabolites were measured in the experiments: 14 amino acids including 13 proteinogenic amino acids and ornithine; hexose (sugars with 6 carbon atoms, e.g. glucose and fructose); 23 acylcarnitines [Cx:y-carn] (with *x *carbon atoms and *y *double bonds), 7 hydroxy-acylcarnitines [Cx:y-OH-carn], 6 dicarboxy-acylcarnitines [Cx:y-DC-carn], and 2 methylated dicarboxy-acylcarnitines variants [Cx:y-M-DC-carn]; 9 sphingomyelins [SM Cx:y] and 5 hydroxy-sphingomyelins [SM Cx:y-OH]; and 87 phosphatidylcholines (PC). These glycerophospholipids are further subdivided with respect to the presence of ester and ether bonds of fatty acid residues with the glycerol moiety. The set contains 36 diacyl-PCs with two esterified fatty acid residues [PC aa Cx:y], 38 acyl-alkyl-PCs with one ether-bond at the sn-2 position [PC ae Cx:y] and 13 lyso-PCs with only one esterified fatty acid residue at the sn-1 or sn-2 position [lysoPC a Cx:y]. Our mass spectometry technology cannot distinguish between the side chains of diacyl-phospholipids. The measured compounds are thus associated with the sum of carbon atoms and double bounds for both fatty acid residues. To ensure log-normality, we compared QQ-plots against normal distributions [45] for both non-logarithmized and logarithmized metabolite concentrations. All distributions were closer to log-normality than to regular normality (not shown), so we logarithmized the metabolite concentrations for the following analysis steps.

### Sensitivity and specificity

In order to objectively evaluate the discrimination between directly and indirectly connected metabolites, we calculated sensitivity and specificity as:

$$\text{sens}:=\frac{\text{TP}}{\text{TP}+\text{FN}},\text{spec}:=\frac{\text{TN}}{\text{TN}+\text{FP}}$$

with TP true positives, FP false positives, TN true negatives, FN false negatives [46].

A metabolite pair is considered true positive if it exhibits a partial correlation above the threshold and has a direct pathway connection; a false positive represents a metabolite pair also above the threshold but with no direct pathway connection; a false negative pair lies below the threshold but does have a direct pathway connection; and finally a true negative pair lies below the threshold and also has no direct pathway connection. The *F*_1 _score was calculated as the harmonic mean of both quantities:

$$F_{1}:=2\cdot \frac{\text{sens}\cdot \text{spec}}{\text{sens}+\text{spec}}$$

### Pathway model

Pathway reactions in the human fatty acid metabolism were drawn from three independent databases: (1) *H. sapiens Recon 1 *from the BiGG databases (confidence score of at least 4) [7], (2) the Edinburgh Human Metabolic Network reconstruction [31] and (3) the KEGG PATHWAY database [29] as of July 2010. A complete list of all curated reactions and the corresponding database identifiers can be found in Additional file 6. The reaction set was subdivided into two groups: (1) Fatty acid biosynthesis reactions which apply to the metabolite classes lyso-PC, diacyl-PC, acyl-alkyl-PC and sphingomyelins. (2) *β*-oxidation reactions representing fatty acid degradation to model reactions between the acyl-carnitines. The *β*-oxidation model consists of a linear chain of C2 degradation steps (C10→C8→C6 etc.).

Fatty acid residues with identical masses, that cannot be distinguished by our mass-spectrometry technology, are merged into a single metabolite in the reaction set. For instance, the polyunsaturated fatty acids C20:4Δ8,11,14,17 from the omega-3 pathway and C20:4 Δ5,8,11,14 from the omega-6 pathway have identical numbers of carbon atoms and double bonds and are thus merged into a single metabolite C20:4.

## Authors' contributions

JK, KS and FJT conceived this data analysis project. TI and JA performed the sample preparation and data acquirement. JK performed the analysis and wrote the primary manuscript. All authors approved the final manuscript.

## Supplementary Material

Additional file 1 **Further results on computer-simulated networks**. Click here for file

Additional file 2 **Effects of genetic variation on GGM calculation**. Click here for file

Additional file 3 **Modularity: Optimized partitioning and weighted calculation**. Click here for file

Additional file 4 **Stability of the GGM with respect to changes in the underlying data set**. Click here for file

Additional file 5 **comparison with low-order partial correlation approaches**. Click here for file

Additional file 6 **Literature-curated pathway model of human fatty acid biosynthesis and degradation**. Click here for file
