# Supplementary material for: Gaussian graphical modeling reconstructs pathway reactions from high-throughput metabolomics data
Source: BMC Syst Biol. 2011 Jan 31;5:21. doi: 10.1186/1752-0509-5-21 (PMC3224437; doi:10.1186/1752-0509-5-21)
Supplement: Additional file 1 — Further results on computer-simulated networks. [file 1752-0509-5-21-S1.PDF]

Additional file 1 –
Further results on computer-simulated networks

Contents

1 Uniqueness of steady states **2**

2 GGM estimation on various first-order kinetics networks **3**

3 Input noise dependence of GGM estimation **6**

4 Enzyme-catalyzed reactions **9**

5 Negative feedback **11**

6 Model download **12**

References **12**

1 Uniqueness of steady states

For all computer simulations used in this study (from both the main manuscript and this supplementary text) it is important to verify that each system only exhibits a single stable steady state. Multistability is an import aspect in many biological systems, cf. e.g. [1, 2, 3] to name but just a few. In our case, however, multiple equilibria could create spurious correlations, possibly leading to misinterpretations of the reconstructed pathway reactions. Therefore we used the ERNEST toolbox [4] to structurally verify uniqueness of a single steady state independent of actual parameter assignments[1].

- All first-order networks in our study show a deficiency of zero [5] and thus cannot exhibit more than one positive steady states.
- For the enzyme-driven networks with reversible Michaelis Menten kinetics and the bimolecular split network (Figure 1G), the deficiency zero theorem does not apply. For these networks, however, the deficiency one theorem [5] applies, and the stoichiometric matrix is *strongly sign determined* (SSD) [6], so the networks do not have the capacity for multiple equilibria.
- The stoichiometry matrix of the cofactor network (Figure 1F) is SSD, so this system also gives rise to a single steady state for any given parameter combination.
- The mixed-inhibition enzyme mechanisms employed in our work (Figure 1D) are known to constitute bistability [2]. However, the two parameter combinations provided in that paper differ in several orders of magnitude for many parameters. Our toy models do not cover such a huge range of parameters and, more importantly, such parameter differences are also not expected to occur in a human population cohort with similar metabolic states (namely fasting). In order to further ensure monostability for our parameter ranges, we let the system run from 1000 different initial states for 100 parameter combinations. Initial values were uniformly drawn between zero and 1000 times the metabolite concentrations of the detected steady state. We did not encounter a single case where a system with identical parameters ended up in a different steady state for a different initial value setting.

[1] all toy models are provided in an ERNEST-compatible format on the paper website at `http://hmgu.de/cmb/ggm`

2 GGM estimation on various first-order kinetics networks

In the following analysis we investigated the performance of GGMs to discriminate direct from indirect pathway interactions on various computer-simulated networks (Figure S1A). These include:

1. Three metabolites connected in a row (*Chain 3*), with all 8 variants of reversible inner reactions and reversible external reactions
2. Five metabolites connected in a row (*Chain 5*), with all 8 variants of reversible inner reactions and reversible external reactions
3. A branching pathway (*Split*), with all 8 variants of reversible inner reactions and reversible external reactions
4. An irreversible feed-forward loop motif (*FFL*), plus one variant with external reactions
5. An irreversible branching and merging motif (*Diamond*), plus one variant with external reactions
6. A densely interconnected network of six player with several subvariants (*Dense*)

For each network we calculated 500 samples, where each sample consists of 1000 simulations with log-normal noise and subsequent partial correlation computation (see Methods). For each of these 500 samples we calculated the discriminatory power according to the area under the ROC curve (AUC, see Methods and Results). A value of 0.5 represents random guessing whereas values near one (zero) indicate perfect (reversely perfect) discrimination. All 500 AUC values for each network are visualized using a boxplot to emphasize variability in the achieved results (Figure S1B).

We observe the following features:

- Networks with reversible reactions show perfect discrimination (except for a few cases in the *Big split, rev, all out* network which we attribute to parameter outliers).
- Irreversible reactions generally impair the discrimination quality. For the straight chains, there is no discrimination at all between directly and indirectly connected metabolites.
- Input and especially output reactions improve the quality and make discrimination possible even for the irreversible straight chains.
- GGMs can delineate intricate relationships as seen in the *FFL* and *Diamond* networks.
- Discrimination works acceptable for all variants of the *Dense* network, but never perfect due to irreversible reactions and missing input/output mechanisms.

Figure S1A

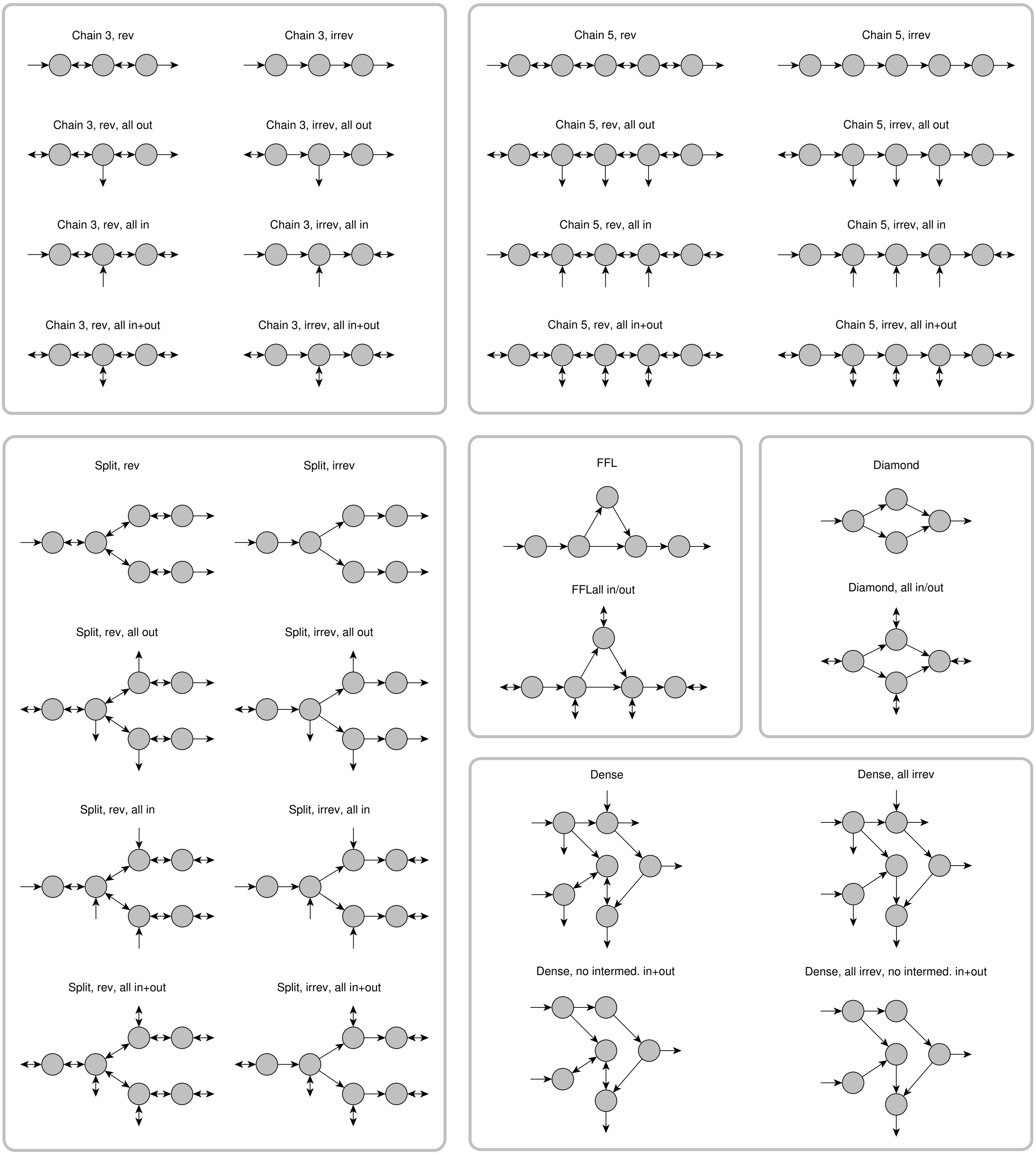
Chain 3, rev
Chain 3, irrev
Chain 3, rev, all out
Chain 3, irrev, all out
Chain 3, rev, all in
Chain 3, irrev, all in
Chain 3, rev, all in+out
Chain 3, irrev, all in+out
Chain 5, rev
Chain 5, irrev
Chain 5, rev, all out
Chain 5, irrev, all out
Chain 5, rev, all in
Chain 5, irrev, all in
Chain 5, rev, all in+out
Chain 5, irrev, all in+out
Split, rev
Split, irrev
Split, rev, all out
Split, irrev, all out
Split, rev, all in
Split, irrev, all in
Split, rev, all in+out
Split, irrev, all in+out
FFL
FFLall in/out
Diamond
Diamond, all in/out
Dense
Dense, all irrev
Dense, no intermed. in+out
Dense, all irrev, no intermed. in+out

Figure S1B

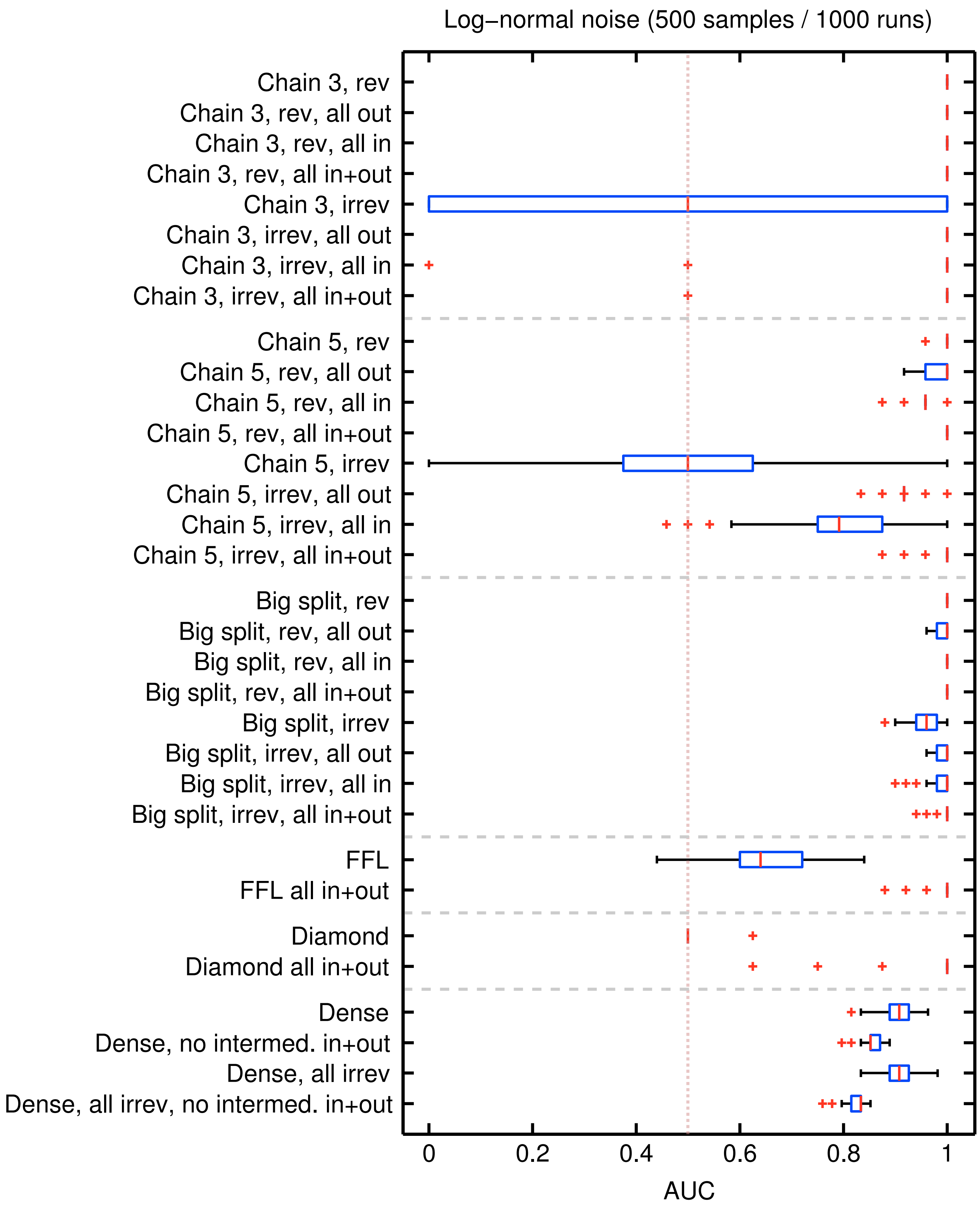
Log−normal noise (500 samples / 1000 runs)
Chain 3, rev
Chain 3, rev, all out
Chain 3, rev, all in
Chain 3, rev, all in+out
Chain 3, irrev
Chain 3, irrev, all out
Chain 3, irrev, all in
Chain 3, irrev, all in+out
Chain 5, rev
Chain 5, rev, all out
Chain 5, rev, all in
Chain 5, rev, all in+out
Chain 5, irrev
Chain 5, irrev, all out
Chain 5, irrev, all in
Chain 5, irrev, all in+out
Big split, rev
Big split, rev, all out
Big split, rev, all in
Big split, rev, all in+out
Big split, irrev
Big split, irrev, all out
Big split, irrev, all in
Big split, irrev, all in+out
FFL
FFL all in+out
Diamond
Diamond all in+out
Dense
Dense, no intermed. in+out
Dense, all irrev
Dense, all irrev, no intermed. in+out
0
0.2
0.4
0.6
0.8
1
AUC

3 Input noise dependence of GGM estimation

We analyzed influences of increasing fluctuation strengths for the input reaction of the first metabolite in all toy networks. Applying the log-normal noise model, we ranged the standard deviation of the underlying normal distribution from 0 to 10 for the input reaction of the first metabolite. Standard deviations for all other reactions were kept constant at a value of 0.5.

To actually quantify the discrimination properties we here used raw partial correlation coefficients rather than analyzing the area under the ROC curve. Figure S1C shows 32 diagrams, one for each toy model. Each diagram plots partial correlation coefficients (y-axis) of directly connected metabolites (blue) and indirectly connected metabolites (red) for increasing values of the input reaction noise strength (x-axis).

In Figure S1D we plotted the differences of medians between PCC values of directly and indirectly connected metabolites as a measure of discriminatory quality. This visualization emphasizes the finding that, especially for the networks that contain further input/output reactions for the remaining metabolites (e.g. *Chain 3, all in/out*), the distance between direct and indirect interactions increases with increasing external noise.

We marked the networks used in Figure 1 A-C from the main manuscript with the letters A, B and C, respectively.

Figure S1C

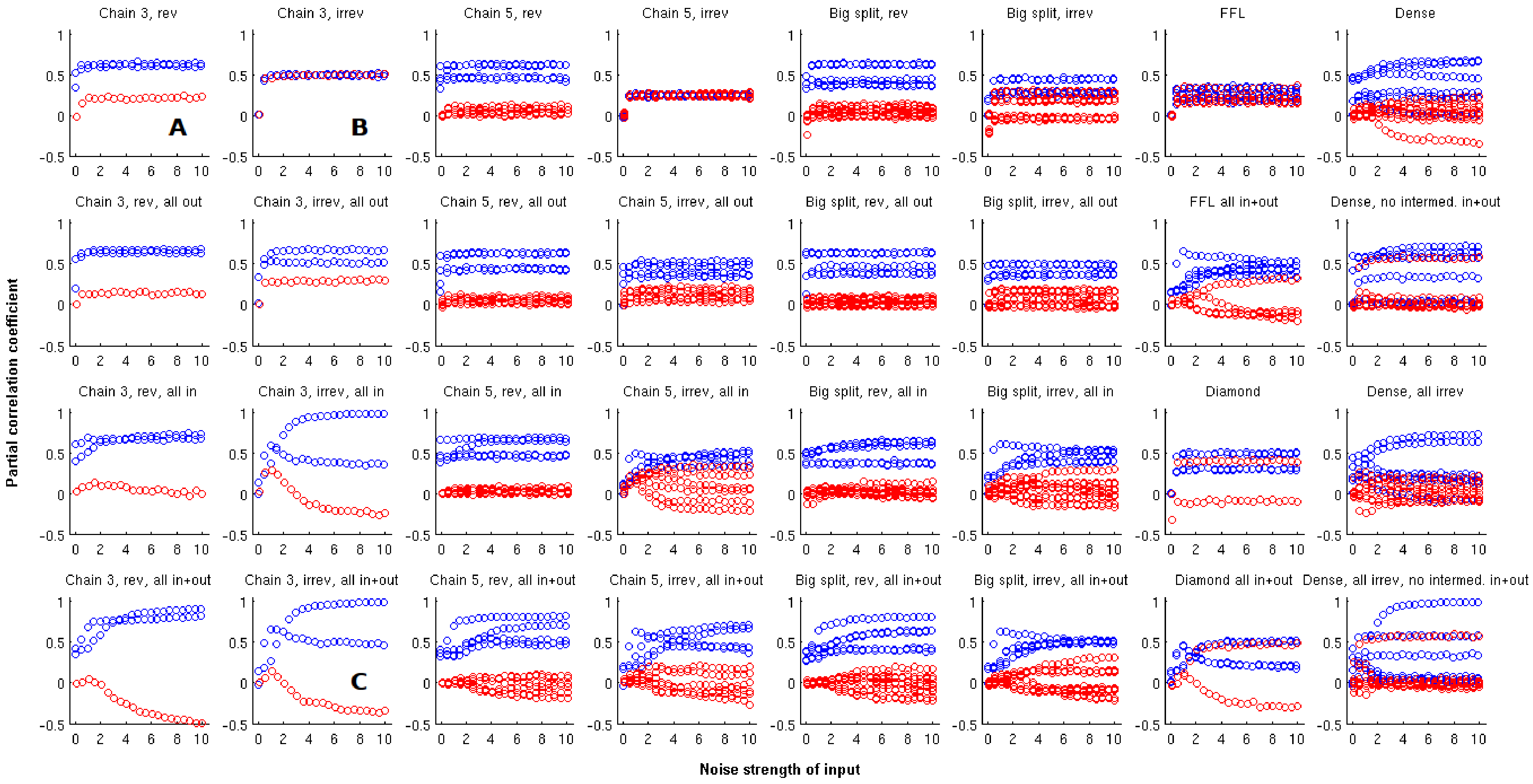

Chain 3, rev
Chain 3, irrev
Chain 5, rev
Chain 5, irrev
Big split, rev
Big split, irrev
FFL
Dense
Chain 3, rev, all out
Chain 3, irrev, all out
Chain 5, rev, all out
Chain 5, irrev, all out
Big split, rev, all out
Big split, irrev, all out
FFL all in+out
Dense, no intermed. in+out
Chain 3, rev, all in
Chain 3, irrev, all in
Chain 5, rev, all in
Chain 5, irrev, all in
Big split, rev, all in
Big split, irrev, all in
Diamond
Dense, all irrev
Chain 3, rev, all in+out
Chain 3, irrev, all in+out
Chain 5, rev, all in+out
Chain 5, irrev, all in+out
Big split, rev, all in+out
Big split, irrev, all in+out
Diamond all in+out
Dense, all irrev, no intermed. in+out
A
B
C
Partial correlation coefficient
Noise strength of input

Figure S1D

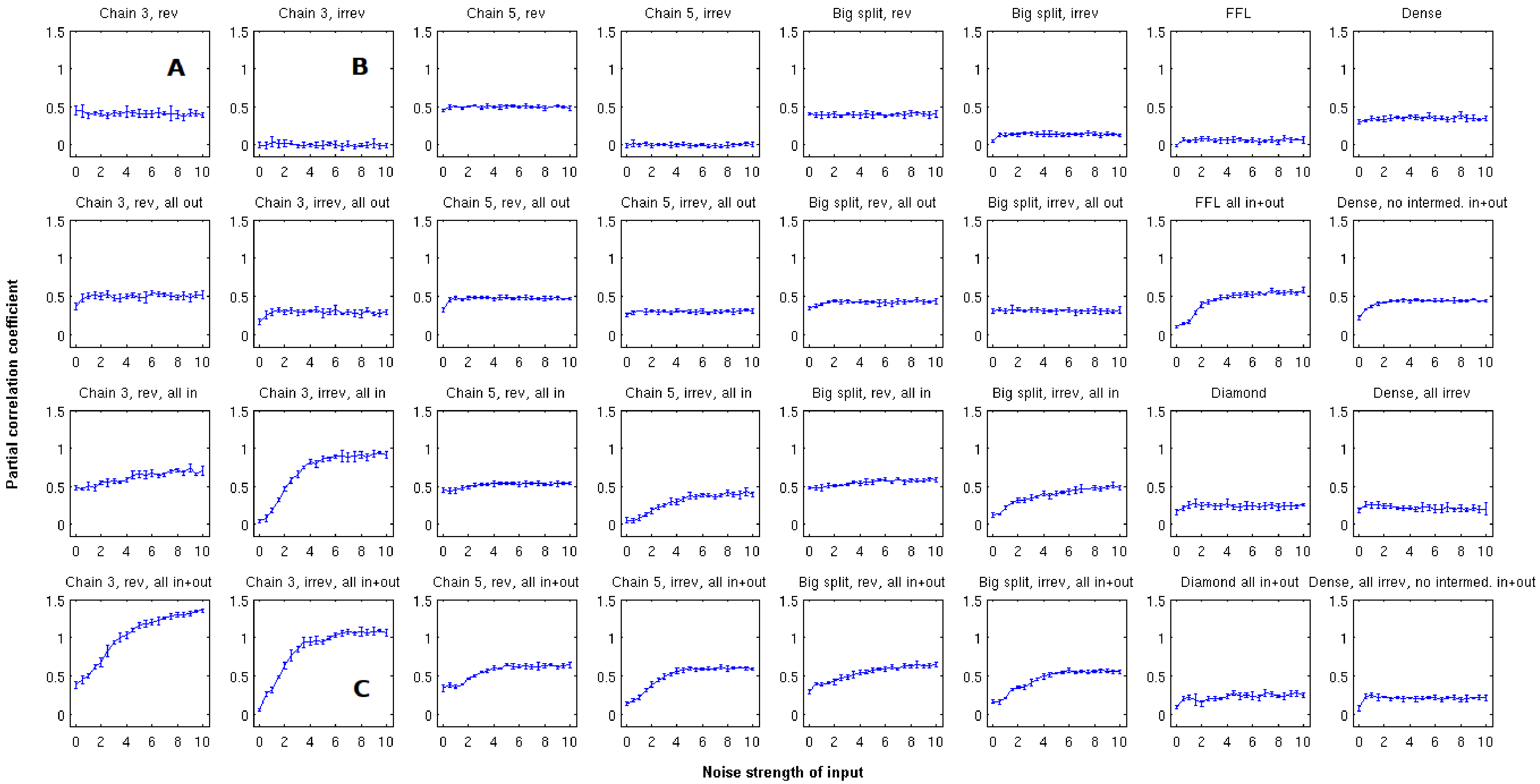
Chain 3, rev
Chain 3, irrev
Chain 5, rev
Chain 5, irrev
Big split, rev
Big split, irrev
FFL
Dense
Chain 3, rev, all out
Chain 3, irrev, all out
Chain 5, rev, all out
Chain 5, irrev, all out
Big split, rev, all out
Big split, irrev, all out
FFL all in+out
Dense, no intermed. in+out
Chain 3, rev, all in
Chain 3, irrev, all in
Chain 5, rev, all in
Chain 5, irrev, all in
Big split, rev, all in
Big split, irrev, all in
Diamond
Dense, all irrev
Chain 3, rev, all in+out
Chain 3, irrev, all in+out
Chain 5, rev, all in+out
Chain 5, irrev, all in+out
Big split, rev, all in+out
Big split, irrev, all in+out
Diamond all in+out
Dense, all irrev, no intermed. in+out
A
B
C
Partial correlation coefficient
Noise strength of input

4 Enzyme-catalyzed reactions

We used the following simple reaction system throughout this analysis.

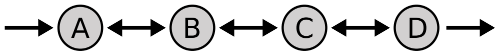

Both internal reaction steps were modeled using reversible Michaelis-Menten kinetics (see Methods section of the main manuscript).

Noise model and parameter dependency

The log-normal noise assumption of cellular rate parameters used in our study can be interpreted as a log-normal variation of V_{max} parameters in the Michaelis-Menten case. Since K_M is supposed to be an intrinsic property of the respective enzyme-substrate interaction, this parameter was kept constant throughout each simulation.

Similar to the first-order case described in the main manuscript, one simulation consists of drawing 5000 parameter sets, calculating a steady state for each parameter sample and subsequent GGM estimation using these 5000 steady states. We performed simulations for (a) different *mean* values of V_{max}^{+}, while V_{max}^{-} was kept constant in order to investigate different degrees of reaction reversibility; (b) different *mean* values of both K_M parameters to introduce different levels of response linearity; (c) different *mean* values of the zeroth-order input reaction carrying A into the system; (d) different levels of noise strengths, i.e. the standard deviation of the underlying log-normal distribution. All parameters means were again set to 1 by default, except for K_M where we choose the mean as 0.01. We only accepted parameter combinations that reach a stable steady state. In contrast to mass-action kinetics, Michaelis-Menten kinetics introduce an upper bound to the reaction rate (namely V_{max}). Specifically, if the constant influx into the system exceeds the net rate from A to B, A will grow infinitely large.

The following figure displays partial correlation coefficients for directly connected (blue) and indirectly connected (red) metabolites for a varying range of parameters:

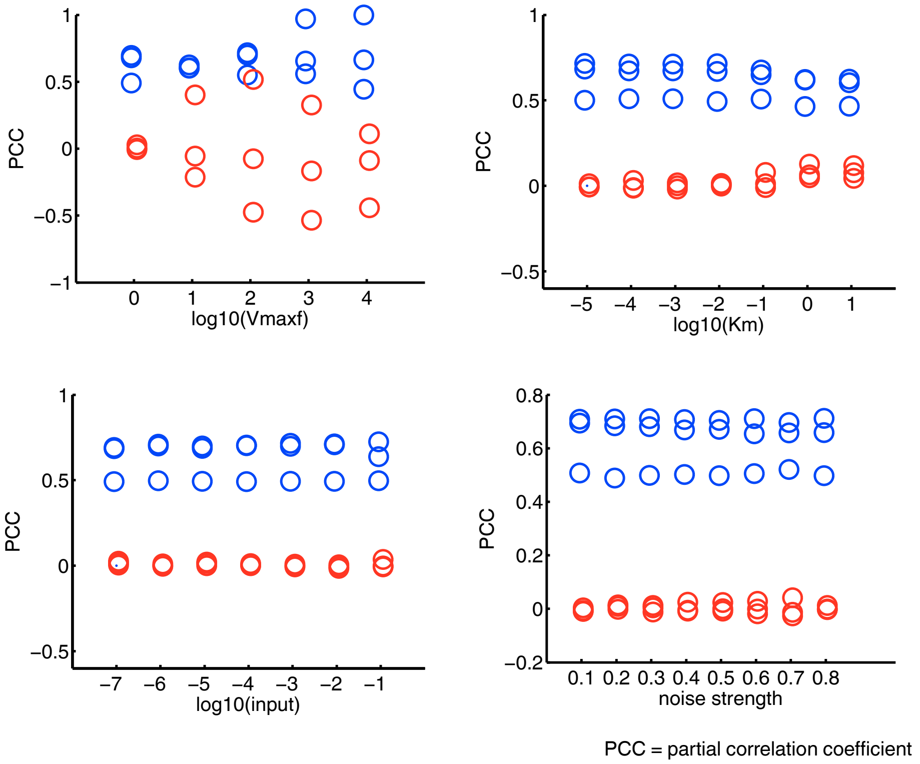

Results:

- (upper left panel) Note that V_{max}^{-} was kept at a constant value of 1, and thus the parameter value in this plot represents the ratio between forward and backward reaction rate. We see that for all parameter values of V_{max}^{+} the GGM theoretically distinguishes direct from indirect interactions. However, if the forward reaction rate exceeds the backward reaction by far (e.g. $\log10(V_{\text{max}}^{+}) = 2$, that is $V_{\text{max}}^{+} = 100$) the discrimination quality is impaired. This is in line with the observation that purely irreversible reactions cannot be distinguished in the mass-action case (see main manuscript). For the cases $\log10(V_{\text{max}}^{+}) = 3$ and 4, unusually high regular Pearson correlations occur (mean overall correlation >0.985), and thus the seemingly improved GGM reconstructions cannot be considered stable for these cases.
- (other panels) No other parameters displayed significant impacts on GGM calculation. This is particularly interesting for the Michaelis constants K_M, which adjust the grade of saturation in the activation curve. Low values in this parameter cause a quick saturation towards the respective V_{max} value. However, since our approach does not investigate actual reaction speeds but rather the steady state levels at equilibrium, the underlying network topology generates a stronger signal in the GGM than enzyme response patterns.

5 Negative feedback

We performed simulations using standard parameter values for enzyme kinetics (see above) with ranging values of the inhibition parameter K_i, once without and once including exchange fluxes for intermediate metabolites:

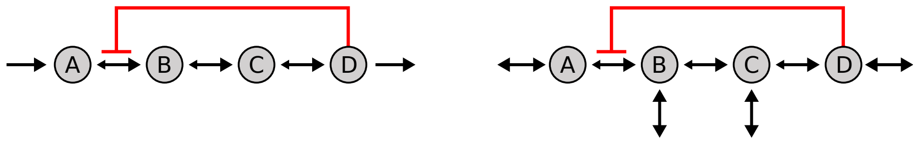

Note that we assume a mixed inhibition model with $K_i = K_{ii}$ (see Methods of the main manuscript). Again, we plotted partial correlation coefficients for directly connected (blue) and indirectly connected (red) metabolites:

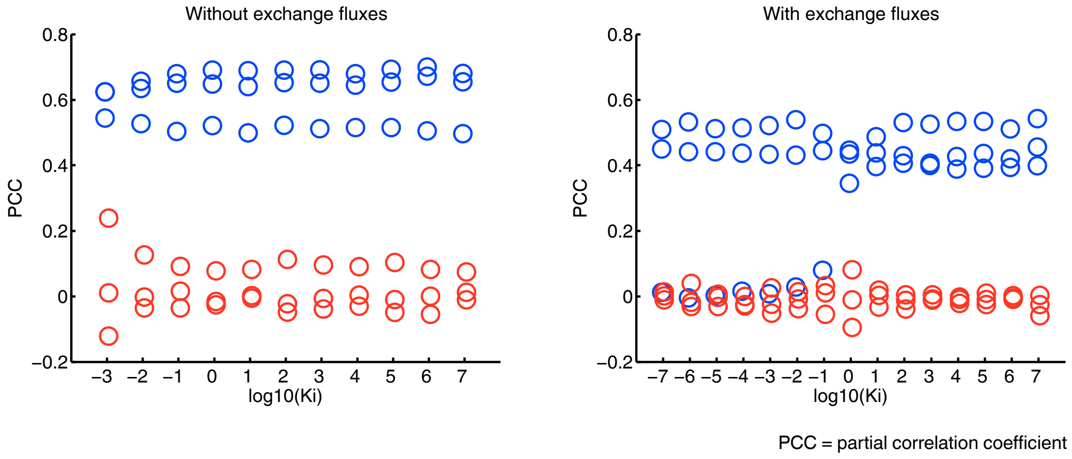

- (left panel) If no exchange fluxes are present, the inhibition strength does not significantly affect discrimination quality. Mass-flow has to be routed through the metabolite chain, independent of any feedback effects. Since we still assumed reversibility of this setup ($V_{\text{max}}^{+} = 2, V_{\text{max}}^{-} = 1$), topological structures can be correctly reconstructed by the GGM. Note that for K_i values below 10^{-3} and our parameter setup, the system reaches steady states only after a very long simulation time and A grows unusually large. We do not expect such situations to occur in a real biological system.
- (right panel) If exchange fluxes are introduced, the inhibitory influence decouples A from the remaining metabolites if K_i falls a certain threshold (here 1). In the plot, we observe one of the blue circles (representing the partial correlation between A and B) reaching noise levels for $K_i = 10^{-1}$ and below. Conclusively, we need to keep in mind that inhibitory feedbacks might impair the reconstruction process and lead to false negative results.

6 Model download

All models used in this paper can be downloaded in various formats from the supplementary website at:

`http://hmgu.de/cmb/ggm`

References

[1] Tyson, J.J., Csikasz-Nagy, A., and Novak, B. The dynamics of cell cycle regulation. *Bioessays*, 24(12):1095–1109, 2002.

[2] Craciun, G., Tang, Y., and Feinberg, M. Understanding bistability in complex enzyme-driven reaction networks. *Proc Natl Acad Sci U S A*, 103(23):8697–8702, 2006.

[3] Huang, S., Guo, Y.P., May, G., and Enver, T. Bifurcation dynamics in lineage-commitment in bipotent progenitor cells. *Dev Biol*, 305(2):695–713, 2007.

[4] Soranzo, N. and Altafini, C. Ernest: a toolbox for chemical reaction network theory. *Bioinformatics*, 25(21):2853–2854, 2009.

[5] Feinberg, M. Chemical reaction network structure and the stability of complex isothermal reactors - i. the deficiency zero and deficiency one theorems. *Chemical Engr. Sci.*, 42:2229–2268, 1987.

[6] Banaji, M., Donnell, P., and Baigent, S. P matrix properties, injectivity, and stability in chemical reaction systems. *SIAM Journal of Applied Mathematics*, 67(6):1523–1547, 2007.
