# Supplementary material for: Gaussian graphical modeling reconstructs pathway reactions from high-throughput metabolomics data
Source: BMC Syst Biol. 2011 Jan 31;5:21. doi: 10.1186/1752-0509-5-21 (PMC3224437; doi:10.1186/1752-0509-5-21)
Supplement: Additional file 2 — Effects of genetic variation on GGM calculation. [file 1752-0509-5-21-S2.PDF]

Additional file 2 –
Effects of genetic variation on GGM calculation

SNP set

In order to assess whether genetic variation affects partial correlation coefficients in the Gaussian graphical model, we added the 15 SNPs that showed genome-wide-significant effects on plasma metabolite concentrations in the GWAS study by Illig et al. [1]. The following table contains the identifiers of these 15 SNPs along with minor allele frequencies (MAF) and protein coding gene loci associated via linkage disequilibrium:

SNP	MAF	Locus
rs174547	30.4%	FADS1
rs2014355	27.7%	ACADS
rs211718	30.5%	ACADM
rs2286963	36.5%	ACADL
rs9393903	24.6%	ELOVL2
rs2216405	18.5%	CPS1
rs7156144	41.4%	PLEKHH1
rs11158519	14.5%	SYNE2
rs168622	37.5%	SPTLC3
rs8396	29.8%	ETFDH
rs7094971	13.5%	SLC16A9
rs2046813	32.2%	ACSL1
rs603424	19.4%	SCD
rs272889	38.5%	SLC22A4
rs541503	37.9%	PHGDH

For further information on these SNPs we refer the reader to Table 1 of the original publication [1].

Calculation

The SNP data was integrated with the metabolomics concentrations by appending 15 further columns to the data set, thus extending the 1020×151 data matrix to a 1020×166 matrix. Due to missing SNP allele information, we had to remove 66 probands from the set, leaving a 954×166 data matrix for further analysis. Next, we calculated full-order partial correlations with and without SNPs on these 954 probands by inversion of the covariance matrix (see main text). Notably, both partial correlations as well as the additive genetic model used in the GWAS are based on linear regression techniques. Therefore conditioning against the SNPs during partial correlation computation precisely removes the genetic effects identified in the GWAS.

Results

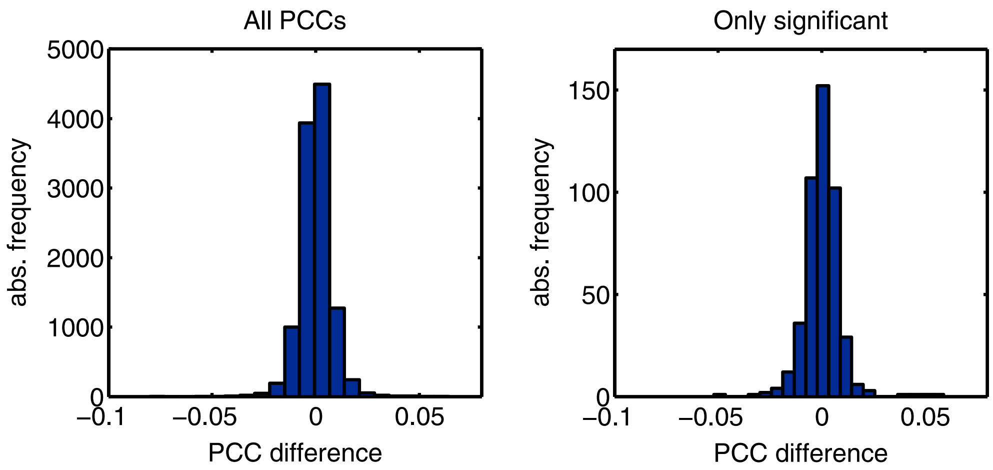

We calculated the differences of partial correlations before and after adding SNPs to the data matrix. Changes are generally small for all partial correlations ($-1.28 \cdot 10^{-5} \pm 8.09 \cdot 10^{-3}$, left histogram), and also when only investigating significant[1] partial correlations ($3.8 \cdot 10^{-4} \pm 1.02 \cdot 10^{-2}$, right histogram). These results indicate that Gaussian graphical models recover intrinsic properties of the metabolic system, and that effects of natural genetic variation are neglectable for our calculations. Causative mutations with strong alterations of enzymatic activity might still influence GGM calculation, but cannot be expected to show a strong effect in our general population data set.

References

[1] Illig, T., Gieger, C., Zhai, G., Römisch-Margl, W., Wang-Sattler, R., Prehn, C., Altmaier, E., Kastenmüller, G., Kato, B.S., Mewes, H.W., Meitinger, T., de Angelis, M.H., Kronenberg, F., Soranzo, N., Wichmann, H.E., Spector, T.D., Adamski, J., and Suhre, K. A genome-wide perspective of genetic variation in human metabolism. *Nat Genet*, 42(2):137–141, 2010.

[1] $\alpha = 0.01$ after Bonferroni correction
