# Supplementary material for: Gaussian graphical modeling reconstructs pathway reactions from high-throughput metabolomics data
Source: BMC Syst Biol. 2011 Jan 31;5:21. doi: 10.1186/1752-0509-5-21 (PMC3224437; doi:10.1186/1752-0509-5-21)
Supplement: Additional file 3 — Modularity: Optimized partitioning and weighted calculation. [file 1752-0509-5-21-S3.PDF]

Additional file 3 –
Modularity: Optimized partitioning and weighted calculation

We already demonstrated that the modularity Q observed for the given metabolite classes is significantly higher than for a random association network (which we obtained by edge reshuffling, see main manuscript). In addition, we were interested in how closely the given metabolite classes represent an optimal partitioning of nodes in the network. Therefore we took a heuristic optimization approach and compared the results with the given six metabolite classes.

Optimization

To find an preferably optimal 6-partitioning of the variables with respect to the modularity and the given GGM, we employed a simulated annealing global optimization approach [1].

The cost function to be minimized was simply defined as the negative modularity value Q. To generate a new candidate solution from an old one we changed the class assignment of one metabolite, but ensuring that no partition contains zero elements. The whole annealing procedure was ran multiple times and the best solution was used[1].

Results

The optimized partitioning achieved a modularity value of $Q = 0.557$ in comparison to the original classes with $Q = 0.488$. The table on the next page shows the original metabolite classes in comparison with the modularity-optimized partitioning. We observe a high similarity between both partitionings, except for the "contamination" of some groups with metabolites from other classes, especially with phospholipid species. These might effects of common fatty acid pools underlying the different lipid species (as discussed in the main manuscript). The introduction of SM C20:2 into the amino acid group can probably be considered a false positive.

Taken together, our findings demonstrate that the GGM is strongly modular with respect to the different metabolite classes, with slight blurring effects in the fatty-acid based lipids.

Weighted calculation

To exclude effects which might have been introduced due to the significance cutoff, we repeated modularity calculation and randomization procedures for the full absolute partial correlation matrix. It is to be noted here that not all degree-preserving properties of edge rewiring in unweighted networks can be transformed to weighted networks. We decided on a neighbor-preserving variant as described in Hartsperger et al. [2].

Modularity values are generally lower than for the undirected case, with $Q = 0.198$ for the original partitioning and $Q = 0.220$ for the optimized variant. Edge rewiring (again with 10^5 samples) yielded $Q = 0.104 \pm 0.004$, resulting in a z-score of $z = 22.49$. Taken together, the results from the weighted analysis can be considered equivalent to the unweighted case.

[1] we used an open-source MATLAB implementation from `http://www.mathworks.com/matlabcentral/fileexchange/10548`

**Original classes**	**Optimized partitioning**
Arg, Gln, Gly, His, Met, Orn, Phe, Pro, Ser, Thr, Trp, Tyr, Val, xLeu	Arg, Gln, Gly, His, Met, Orn, Phe, Pro, Ser, Thr, Trp, Tyr, Val, xLeu, SM C20:2
C0:0, C10:0, C10:1, C10:2, C12:0, C12-DC, C12:1, C14:0, C14:1, C14:1-OH, C14:2, C14:2-OH, C16:0, C16:1, C16:1-OH, C16:2-OH, C18:0, C18:1, C18:2, C2:0, C3:0, C3:0-DC / C4:0-OH, C5:0-OH, C4:0, C4:1-DC / C6:0, C5:0, C6:OH, C5-M-DC, C5:1, C5:1-DC, C6:1, C7:0-DC, C8:0, C8:1, C9:0	C0:0, C10:0, C10:1, C10:2, C12:0, C12-DC, C12:1, C14:0, C14:1, C14:1-OH, C14:2, C14:2-OH, C16:0, C16:1, C16:1-OH, C16:2-OH, C18:0, C18:1, C18:2, C2:0, C3:0, C3:0-DC / C4:0-OH, C5:0-OH, C4:0, C4:1-DC / C6:0, C5:0, C6:OH, C5-M-DC, C5:1, C5:1-DC, C6:1, C7:0-DC, C8:0, C9:0
lysoPC a C14:0, lysoPC a C16:0, lysoPC a C16:1, lysoPC a C17:0, lysoPC a C18:0, lysoPC a C18:1, lysoPC a C18:2, lysoPC a C20:3, lysoPC a C20:4, lysoPC a C24:0, lysoPC a C26:1, lysoPC a C28:0, lysoPC a C28:1	PC aa C24:0, PC aa C26:0, PC aa C42:4, PC ae C30:2, lysoPC a C14:0, lysoPC a C16:0, lysoPC a C17:0, lysoPC a C18:2, lysoPC a C20:3, lysoPC a C20:4, lysoPC a C24:0, lysoPC a C26:1, lysoPC a C28:0, lysoPC a C28:1
PC aa C24:0, PC aa C26:0, PC aa C28:1, PC aa C30:0, PC aa C32:0, PC aa C32:1, PC aa C32:2, PC aa C32:3, PC aa C34:1, PC aa C34:2, PC aa C34:3, PC aa C34:4, PC aa C36:0, PC aa C36:1, PC aa C36:2, PC aa C36:3, PC aa C36:4, PC aa C36:5, PC aa C36:6, PC aa C38:0, PC aa C38:3, PC aa C38:4, PC aa C38:5, PC aa C38:6, PC aa C40:1, PC aa C40:2, PC aa C40:3, PC aa C40:4, PC aa C40:5, PC aa C40:6, PC aa C42:0, PC aa C42:1, PC aa C42:2, PC aa C42:4, PC aa C42:5, PC aa C42:6	C8:1, PC aa C30:0, PC aa C32:0, PC aa C32:1, PC aa C32:2, PC aa C32:3, PC aa C34:1, PC aa C34:2, PC aa C34:3, PC aa C34:4, PC aa C36:1, PC aa C36:2, PC aa C36:3, PC aa C36:4, PC aa C36:5, PC aa C36:6, PC aa C38:3, PC aa C38:4, PC aa C38:5, PC aa C38:6, PC aa C40:1, PC aa C40:2, PC aa C40:3, PC aa C40:4, PC aa C40:5, PC aa C40:6, PC aa C42:2, PC aa C42:5, PC aa C42:6, PC ae C38:0, PC ae C40:0, PC ae C42:0, lysoPC a C16:1, lysoPC a C18:0, lysoPC a C18:1
PC ae C30:0, PC ae C30:2, PC ae C32:1, PC ae C32:2, PC ae C34:0, PC ae C34:1, PC ae C34:2, PC ae C34:3, PC ae C36:0, PC ae C36:1, PC ae C36:2, PC ae C36:3, PC ae C36:4, PC ae C36:5, PC ae C38:0, PC ae C38:1, PC ae C38:2, PC ae C38:3, PC ae C38:4, PC ae C38:5, PC ae C38:6, PC ae C40:0, PC ae C40:1, PC ae C40:2, PC ae C40:3, PC ae C40:4, PC ae C40:5, PC ae C40:6, PC ae C42:0, PC ae C42:1, PC ae C42:2, PC ae C42:3, PC ae C42:4, PC ae C42:5, PC ae C44:3, PC ae C44:4, PC ae C44:5, PC ae C44:6	PC aa C36:0, PC aa C38:0, PC aa C42:0, PC aa C42:1, PC ae C30:0, PC ae C32:1, PC ae C32:2, PC ae C34:0, PC ae C34:1, PC ae C34:2, PC ae C34:3, PC ae C36:0, PC ae C36:1, PC ae C36:2, PC ae C36:3, PC ae C36:4, PC ae C36:5, PC ae C38:1, PC ae C38:2, PC ae C38:3, PC ae C38:4, PC ae C38:5, PC ae C38:6, PC ae C40:1, PC ae C40:3, PC ae C40:4, PC ae C40:5, PC ae C40:6, PC ae C42:1, PC ae C42:2, PC ae C42:3, PC ae C42:4, PC ae C42:5, PC ae C44:3, PC ae C44:4, PC ae C44:5, PC ae C44:6
SM (OH) C14:1, SM (OH) C16:1, SM (OH) C22:1, SM (OH) C22:2, SM (OH) C24:1, SM C16:0, SM C16:1, SM C18:0, SM C18:1, SM C20:2, SM C24:0, SM C24:1, SM C26:0, SM C26:1	PC aa C28:1, PC ae C40:2, SM (OH) C14:1, SM (OH) C16:1, SM (OH) C22:1, SM (OH) C22:2, SM (OH) C24:1, SM C16:0, SM C16:1, SM C18:0, SM C18:1, SM C24:0, SM C24:1, SM C26:0, SM C26:1

References

[1] Kirkpatrick, S., Gelatt, C.D., and Vecchi, M.P. Optimization by simulated annealing. *Science*, 220(4598):671–680, 1983.

[2] Hartsperger, M.L., Blöchl, F., Stümpflen, V., and Theis, F.J. Structuring heterogeneous biological information using fuzzy clustering of k-partite graphs. *BMC Bioinformatics*, 11:522, 2010.
