# Supplementary material for: Gaussian graphical modeling reconstructs pathway reactions from high-throughput metabolomics data
Source: BMC Syst Biol. 2011 Jan 31;5:21. doi: 10.1186/1752-0509-5-21 (PMC3224437; doi:10.1186/1752-0509-5-21)
Supplement: Additional file 4 — Stability of the GGM with respect to changes in the underlying data set. [file 1752-0509-5-21-S4.PDF]

Additional file 4 – Stability of the GGM with respect to changes in the underlying data sets

S1

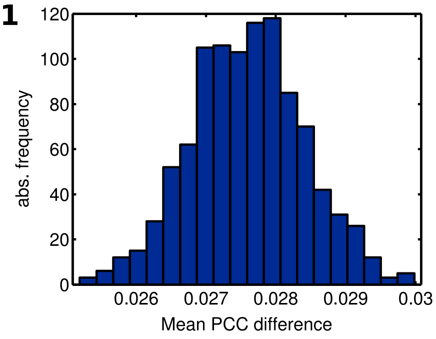

S2

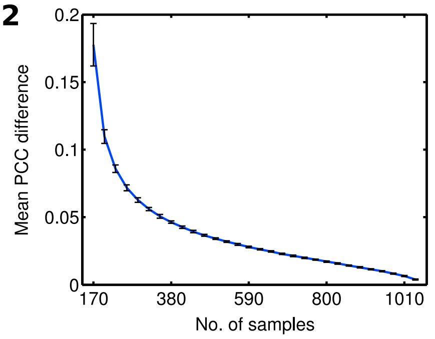

S1: Mean differences of partial correlation coefficients from the values calculated for the original dataset, obtained by generating 1000 bootstrap samples and then calculating the mean absolute distance to the original correlations. Deviations from the original values are relatively low, indicating for a high stability of PCC with respect to changes in the dataset. Note that we observe a slightly smaller mean difference when only taking into account the significant PCCs, indicating a higher stability of stronger signals in the data (mean difference 0.0222).

S2: Mean differences from the original dataset for varying sample sizes. For each tested dataset size, the respective number of samples was randomly drawn from the original dataset 100 times (standard deviations are plotted in black). Even for smaller sample sizes than 1020 we would have obtained a reasonably good approximation of the GGM. For instance, for $N = 530$ we observe a mean PCC difference of 0.03.
