# Supplementary material for: Gaussian graphical modeling reconstructs pathway reactions from high-throughput metabolomics data
Source: BMC Syst Biol. 2011 Jan 31;5:21. doi: 10.1186/1752-0509-5-21 (PMC3224437; doi:10.1186/1752-0509-5-21)
Supplement: Additional file 5 — comparison with low-order partial correlation approaches. [file 1752-0509-5-21-S5.PDF]

Additional file 5 – comparison with low-order partial correlation approaches

We calculated first-, second- and third-order partial correlation coefficients according to de la Fuente et al. [1] with the software packages provided at

`http://mendes.vbi.vt.edu/tiki-index.php?page=Software`

In the following two sections, we discuss the performance of these low-order partial correlations in comparison to the GGM, i.e. $(n\text{-}2)th$-order partial correlations, on both toy examples and the real metabolomics data.

Toy examples

We will exemplarily discuss the case of first-order partial correlations. The reconstruction results will be correct for two given nodes whenever the removal of one other node is sufficient to separate the two nodes in the underlying graph. Removing nodes from the network is the graphical depiction of conditioning against variables in the underlying statistical dependence structure. For a detailed description we refer the reader to Castelo et al. [2]. If multiple paths through the graph are possible between two nodes, conditioning against just one further node cannot be enough to rule out indirect effects. The situation is further illustrated with the following two reaction systems:

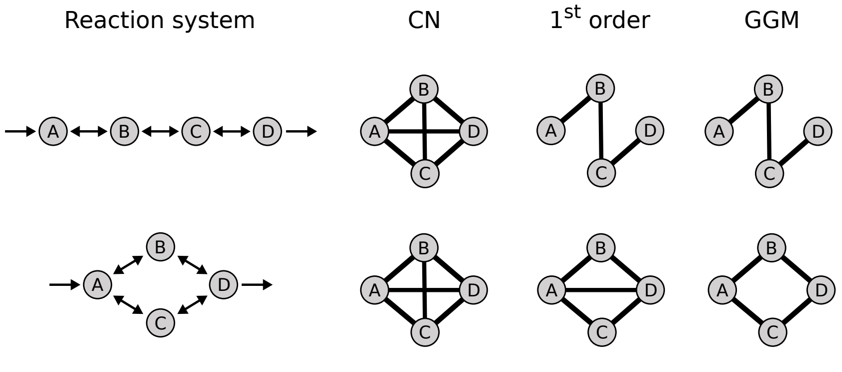

CN = correlation network, 1st order = first-order partial correlations, GGM = Gaussian graphical model

For the first network both first-order partial correlations and the GGM (in this case identical to second-order partial correlations) correctly reconstruct the network topology. For instance, A and D can be separated by conditioning on either B or C (or both). For the second network, however, removing just one node from the network is not sufficient to separate the indirectly connected nodes, and thus first-order partial correlations fail to reconstruct the correct topology.

The same principles obviously hold true for higher-order partial correlations. In general, in order for n-th-order partial correlations to work correctly, any

indirectly connected pair of nodes in the underlying graph must be separable by the removal of n nodes. Since we do not know the true dependency structure, the usage of GGMs is a simple and unbiased reconstruction approach; given that they can be properly estimated from the given amount of samples in the data set.

Discrimination performance on the metabolomics data set

Next we investigated whether low-order partial correlations discriminate directly connected from indirectly connected metabolites in our real data set. Comparison of partial correlation coefficients against pathway distances and sensitivity/specificity analysis was performed analogously to the results presented in Figure 5 of the main manuscript. We again constructed networks from correlation matrices by cutting at a significance level of $\alpha = 0.01$ after Bonferroni correction, and then calculated the F_1 measure as a tradeoff between sensitivity and specificity.

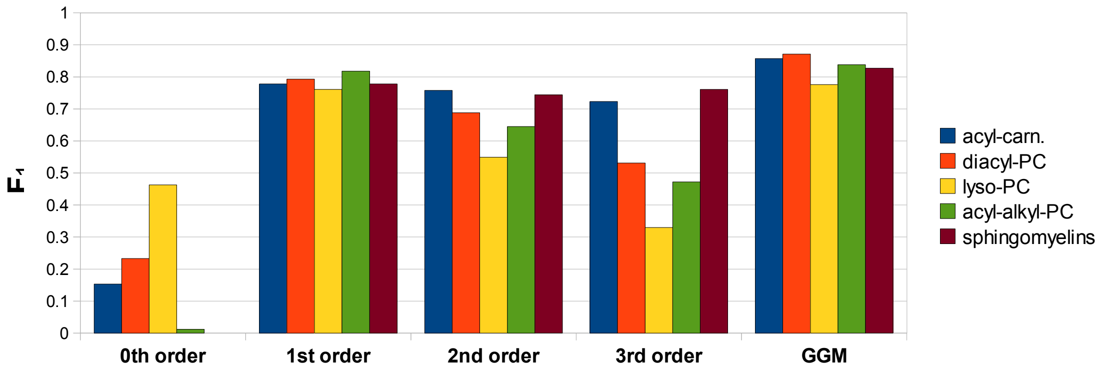

Regular Pearson correlations (0th order) perform poorly in discriminating between direct and indirect interactions, as expected. Surprisingly, first-order partial correlations perform equally well as the full-order GGM network. This result suggests that there exists just one path between two given metabolites for many cases, a finding compatible with the simple structures of fatty acid metabolism (see main manuscript).

The explanation for the poor performance of both second- and third-order partial correlations in this data set remains to be unraveled, since both generally worked in toy systems (not shown, but analogous to the analysis above). Nevertheless, all variants of low-order partial correlations should be considered as alternatives to full-order GGMs for further studies on metabolomics data.

The following table lists sensitivity and specificity for all methods in addition to the F_1 score.

0th order			1st order			2nd order			3rd order			GGM		
sens	spec	F_1	sens	spec	F_1	sens	spec	F_1	sens	spec	F_1	sens	spec	F_1
1.00	0.08	0.15	0.77	0.79	0.78	0.65	0.92	0.76	0.59	0.94	0.72	0.82	0.89	0.86
1.00	0.13	0.23	0.72	0.88	0.79	0.53	0.97	0.69	0.36	1.00	0.53	0.81	0.94	0.87
1.00	0.30	0.46	0.80	0.73	0.76	0.40	0.88	0.55	0.20	0.95	0.33	0.80	0.75	0.78
1.00	0.01	0.01	0.83	0.81	0.82	0.48	0.97	0.65	0.31	0.99	0.47	0.76	0.94	0.84
1.00	0.00	0.00	1.00	0.64	0.78	0.67	0.84	0.74	0.67	0.89	0.76	1.00	0.71	0.83

References

[1] de la Fuente, A., Bing, N., Hoeschele, I., and Mendes, P. Discovery of meaningful associations in genomic data using partial correlation coefficients. *Bioinformatics*, 20(18):3565–3574, 2004.

[2] Castelo, R., Roverato, A., and Chickering, M. A robust procedure for gaussian graphical model search from microarray data with p larger than n. *Journal of Machine Learning Research*, 7:2006, 2006.
